# Effect of labour income on the optimal bankruptcy problem

**DOI:** 10.1007/s10479-023-05166-z

**Published:** 2023-01-22

**Authors:** Guodong Ding, Daniele Marazzina

**Affiliations:** Department of Mathematics, Politecnico di Milano, 20133 Milano, Italy

**Keywords:** Power utility optimization, Bankruptcy stopping time, Consumption-portfolio-leisure controls, Legendre–Fenchel transform, Variational inequalities

## Abstract

In this paper we deal with the optimal bankruptcy problem for agents who can optimally allocate their consumption rate, the amount of capital invested in the risky asset, as well as their leisure time. In our framework, the agents are endowed by an initial debt, and they are required to repay their debt continuously. Declaring bankruptcy, the debt repayment is exempted at the cost of a wealth shrinkage. We implement the duality method to solve the problem analytically and conduct a sensitivity analysis to the bankruptcy cost and benefit parameters. Introducing the flexible leisure/working rate, and therefore the labour income, into the bankruptcy model, we investigate its effect on the optimal strategies.

**Supplementary Information:**

The online version contains supplementary material available at 10.1007/s10479-023-05166-z.

## Introduction

In this paper, we study an optimal stopping time problem, in which agents decide their consumption-portfolio-leisure strategy as well as the optimal bankruptcy time. Their utility is described by a power function concerning both consumption and leisure rates. The sum of labour and leisure rates is assumed to be equal to a constant $\bar{L}$. The labour rate is lower bounded by a positive constant $\bar{L}-L,\,L>0,$ to retain the employment state. Relating to the labour rate, the agent earns the labour income with a fixed wage rate. By determining the continuous and stopping regions of the corresponding stopping time problem, we prove that the optimal bankruptcy time is the first hitting time of the wealth process downward to a critical wealth boundary.

The idea is directly inspired by Jeanblanc et al. (2004), in which a stochastic control model is constructed to quantify the benefit of filing consumer bankruptcy in the perspective of complete debt erasure. Their research is a response to the sharp growth in bankruptcy cases between 1978 and 2003 due to the promulgation of the 1978 Bankruptcy Reform Act in American. The Act introduced two kinds of consumer bankruptcy mechanisms, which are reflected in its Chapter 7 and Chapter 13 separately: debtors following Chapter 7 to file bankruptcy are granted the debt exemption, but must undertake the liquidation of non-exempt assets. Alternatively, the mechanism in Chapter 13 adopts the reorganization procedure instead of the liquidation. Debtors are permitted to retain assets, but the debt is required to be reorganized and paid continuously from future revenues. The statistical data shows that filing bankruptcy under Chapter 7 predominates in all consumer bankruptcy cases (1,156,274 out of 1,625,208 cases in 2003, accounting for 72%).^1^ Following Jeanblanc et al. (2004), an affine loss function, $\alpha (X(\tau )-F)$, is established to deal with both the fixed and variable costs of filing bankruptcy, which corresponds to the mathematical description for the bankruptcy mechanism under Chapter 7. Here $X(\tau )$ is the wealth level at the moment of bankruptcy $\tau$, and *F* represents the fixed cost of bankruptcy. Therefore, the bankruptcy option reduces the wealth from $X(\tau )$ to $X(\tau ^+)=\alpha (X(\tau )-F)$, with a drop in wealth equal to $F+(1-\alpha )(X(\tau )-F)=(1-\alpha )X(\tau )+\alpha F$. The loss proportion $(1-\alpha )$ of the remaining wealth after the bankruptcy liquidation, $X(\tau )-F$, is related, for example, to taxes.

Compared to Jeanblanc et al. (2004), we make an extension in two aspects: firstly, a new control variable, the leisure rate, is inserted for a more realistic consideration: decreasing the leisure rate, the agent earns a larger labour income. For the introduction of the leisure as a control variable into optimal stopping time problems, the reader can refer to Choi et al. (2008) and Farhi and Panageas (2007), where authors studied the optimal retirement -from labour- model regarding the consumption-portfolio-leisure strategy. Different from these two researches, we consider the stopping time concerning the bankruptcy issue rather than the retirement: while the optimal retirement is the first hitting time of the wealth process to an upper critical wealth boundary (Barucci & Marazzina, 2012; Choi et al., 2008; Farhi & Panageas, 2007), the optimal bankruptcy is related to a lower boundary. This extension permits us to study the impact of the disutility from full work on the bankruptcy option. Secondly, in order to deal with a utility from consumption and leisure rate, we implement a different method from Jeanblanc et al. (2004), where the utility of the agent only depends on her consumption, solving the optimal problem with the duality method instead of the dynamic programming method, to deduce the solution analytically, as in Barucci and Marazzina (2012) and Choi et al. (2008). We would like to stress that in this work we deal with the duality method applied to intertemporal consumption; for terminal utility problem the reader can refer, for example, to Barucci et al. (2021), Colaneri et al. (2021) and Nicolosi et al. (2018). The duality method throughout this paper can be summarized into four steps. We first tackle the post-stopping time problem to deduce a closed form of the corresponding value function. Then we apply the convex dual transform to the utility function and to the value function of the post-bankruptcy time problem obtained in the first step. Afterwards, we construct the duality between the optimal control problem and the individual’s shadow price problem, by the aid of the liquidity and budget constraints and the dual transforms acquired before. Finally, we cast the dual shadow price problem as a free boundary problem, which leads to a system of variational inequalities and enables us to solve it analytically. The methodology discussed here refers to He and Pagès (1993) and Karatzas and Wang (2000): in Karatzas and Wang (2000) authors applied the duality method to solve a discretionary stopping time problem explicitly, while in He and Pagès (1993) authors used the duality approach to link the individual’s shadow price problem with the optimal control problem and investigated the impact of the liquidity constraint on the optimization.

Other related literatures are Karatzas et al. (1997), where authors studied the general optimal control problem involving the consumption and investment, and offered the solution in a closed-form (Sethi et al., 1995), where a general continuous-time consumption and portfolio decision problem with a recoverable bankruptcy option is considered; and Bellalah et al. (2019) where authors address the role of labour earnings in optimal asset allocation.

In the numerical results part, we first of all conduct the sensitivity analysis with respect to the key bankruptcy parameters *F*, $\alpha$ and *d*. The fixed toll *F* and $(1-\alpha )$ can be treated as the fixed and flexible bankruptcy costs; the debt *d* is the continuous-time debt repayment. As already said, the optimal bankruptcy corresponds to the first hitting time of the wealth process of a downward boundary, the bankruptcy wealth threshold. This threshold, as a function of the debt repayment *d*, is an increasing curve. The rationale of this result is the following: a heavier debt repayment, in fact, implies that the benefit of bankruptcy becomes more attractive, therefore inducing the agent to take a higher threshold to make the bankruptcy requirement more accessible to enjoy the debt exemption easily. Similar results hold true if we consider the bankruptcy threshold as a function of $\alpha$, i.e., the proportion of wealth after the bankruptcy liquidation: a lower value of $\alpha$ indicates a higher flexible cost (a higher value of $(1-\alpha )$) and pushes the agent to set a lower wealth level to avoid suffering the bankruptcy. Our numerical results also show the non-monotonic relationship between the bankruptcy wealth threshold and *F*; this is due to the role of *F*, which is not only the fixed cost of bankruptcy, but, according to the model in Jeanblanc et al. (2004), in order to make the problem feasible, it also has an important role as liquidity constraint in the pre-bankruptcy period. Moreover, comparing the optimal control policies between the model with and without the bankruptcy option, we find that the optimal consumption-portfolio-leisure policies when this option is available dominate the ones without it before the bankruptcy. Whereas, after declaring bankruptcy, the agent suffers the wealth shrinkage and prefers to invest less in the risky asset for the needs of obtaining utility from consumption and leisure. In addition, we also study the impact of introducing the leisure rate as a second control variable, in order to investigate the influence of labour income. The numerical result indicates that the optimal consumption and portfolio policies with the flexible leisure option always prevail over the corresponding policies of the model with a full leisure rate, and therefore without labour income, as expected.

The paper is organized as follows. Section 2 formulates the corresponding optimization problem, and provides the financial market setting. Section 3 offers the value function of post-bankruptcy problem and its Legendre–Fenchel transform. In Section 4, we construct the duality between the optimal control problem with the individual’s shadow price, and obtain a free boundary problem which endows us the closed-form optimal solutions. Section 5 presents the sensitivity analysis of the bankruptcy wealth threshold to main parameters. Finally, Sect. 6 concludes. Most of the proofs and computations are reported in the online appendix.

## Problem formulation

### Financial market

We first introduce the financial market assumptions. Based on the mutual fund theorem from Karatzas et al. (1997), we consider only one risky asset which dynamics follows a Geometric Brownian Motion with constant drift and diffusion coefficients. The agent faces two investment opportunities: the investment in the money market, which endows her a fixed and positive interest rate $r>0$, and the risky asset, which dynamically evolves according to the stochastic differential equation (SDE)

2.1 $$\begin{aligned} {\left\{ \begin{array}{ll} dS(t)=\mu S(t)dt+\sigma S(t)dB(t),\\ S(0)=S_{0}. \end{array}\right. } \end{aligned}$$

*B*(*t*) denotes a standard Brownian motion on the filtered probability space $(\Omega ,\mathcal {F},\mathbb {P})$, $\mathbb {F}\!\!=\!\!\{\mathcal {F}_{t},t\!\!\ge \!\! 0 \}$ is the augmented natural filtration on this Brownian motion, and $S_{0}$ represents the initial stock price, which is assumed to be a positive constant. Since the drift and diffusion terms $\mu$ and $\sigma$ are constants, there exists a unique solution to the SDE (2.1), $S(t)=S_{0}e^{\left( \mu -\frac{1}{2}\sigma ^{2}\right) t+\sigma B(t)}$.

Then referring to Karatzas and Shreve (1998), we introduce the state-price density process as $H(t)\triangleq \xi (t)\tilde{Z}(t)$, with $\xi (t)$, $\tilde{Z}(t)$, the discount process and an exponential martingale, respectively defined as

$$\begin{aligned} {\left\{ \begin{array}{ll} \xi (t)\triangleq e^{-rt}, \quad \text{ with }\quad \xi (0)=1,\\ \tilde{Z}(t)\triangleq e^{-\frac{1}{2}\theta ^{2}t-\theta B(t)}, \quad \text{ with }\quad \tilde{Z}(0)=1. \end{array}\right. } \end{aligned}$$

Moreover, $\theta \triangleq \frac{\mu -r}{\sigma }$ stands for the market price of risk, that is, the Sharpe-Ratio. Since the exponential martingale $\tilde{Z}(t)$ is, in fact, a $\mathbb {P}$-martingale, and both the number of risky assets and the dimension of the driving Brownian motions are equal to one, the financial market $\mathcal {M}$ defined with the above setting, $\mathcal {M}=\{(\Omega ,\mathcal {F},\mathbb {P}),B,r,\mu ,\sigma ,S_{0}\}$, is standard and complete, based on the result from Karatzas and Shreve (1998, Section 1.7, Definition 7.3). Additionally, we can define an equivalent martingale measure through $\tilde{\mathbb {P}}(A)\triangleq \mathbb {E}\left[ \tilde{Z}(t)\mathbb {I}_{\{A\}}\right]$, $\forall A\in \mathcal {F}_{t}$. Then based on the Girsanov Theorem, we can get a standard Brownian motion under the $\tilde{\mathbb {P}}$ measure as

2.2 $$\begin{aligned} \tilde{B}(t)\triangleq B(t)+\theta t,\quad \forall t\ge 0. \end{aligned}$$

### The optimization problem

The agent optimally chooses three control variables in the optimization: the consumption rate, the amount of money allocated in the risky asset and the leisure rate, which are denoted as *c*(*t*), $\pi (t)$ and *l*(*t*), respectively. The sum of the labour and leisure rate is constant and equals $\bar{L}$. Therefore, the working rate at time *t* is $(\bar{L}-l(t))$ that enables the agent to earn a wage of $w(\bar{L}-l(t))$, where $w>0$ represents the constant wage rate. Obviously, the condition $0\le l(t)\le \bar{L}$ must be imposed for positive labour income. Furthermore, a realistic constraint is introduced into the model, that is, the working rate should be lower bounded by a positive constant, $\bar{L}-L$, with $L>0$, for the sake of retaining the employment state.

Following Jeanblanc et al. (2004), an affine loss function is introduced to deal with fixed and variable costs of filing bankruptcy. Let $\tau$ denote the bankruptcy time, the agent is obliged to repay continuously a positive fixed debt *d* until the stopping time $\tau$, whereas this debt obligation is exempted after declaring bankruptcy, but with the fixed cost $F>0$ and the variable cost $(1-\alpha )$, with $\alpha \in (0,1)$. In more detail, the agent needs to pay a fixed toll *F* once for all at the time $\tau$, and the $(1-\alpha )$ proportion of the remaining wealth, which is related to the social cost, time cost and taxes cost of declaring bankruptcy. Therefore, the agent is able to keep the amount $\alpha (X(\tau )-F)$ of wealth for the consumption and investment after bankruptcy. For the purpose of making sure that the agent is capable of affording the bankruptcy, the wealth level is required to cover the cost before the stopping time $\tau$, that is, $X(t)\ge F+\eta$, $\forall t\in [0,\tau ]$, where $\eta$ is a small non-negative constant to guarantee that there is still a few amounts of wealth left even after the liquidation. The bankruptcy mechanism described above entails the wealth process *X*(*t*) to satisfy the following SDE

$$\begin{aligned} {\left\{ \begin{array}{ll} X(0)=x ,\\ dX(t)=\left[ rX(t)+\pi (t)\left( \mu -r\right) -c(t)+w(\bar{L}-l(t))-d\right] dt+\sigma \pi (t)dB(t),&{}t\le \tau ,\\ X(\tau ^{+})=\alpha (X(\tau )-F),\\ dX(t)=\left[ rX(t)+\pi (t)\left( \mu -r\right) -c(t)+w(\bar{L}-l(t))\right] dt+\sigma \pi (t)dB(t),&{}t>\tau . \end{array}\right. } \end{aligned}$$

Furthermore, we assume that the agent’s preference is described by a power utility function of consumption and leisure rate

2.3 $$\begin{aligned} u(c,l)=\frac{\left( c^{\delta }l^{1-\delta }\right) ^{1-k}}{\delta (1-k)},\quad 0<\delta <1, \quad k>1. \end{aligned}$$

Setting $k>1$ makes the mixed second partial derivative negative,

$$\begin{aligned} \frac{\partial ^{2}u(c,l)}{\partial c\partial l}=(1-k)(1-\delta )l^{(1-k)(1-\delta )-1}c^{\delta (1-k)-1}<0, \end{aligned}$$

which clarifies that consumption and leisure are substitute goods. The following lemma introduces the convex dual transform of the function *u*(*c*, *l*)

2.4 $$\begin{aligned} \tilde{u}(y)\triangleq \sup _{c\ge 0,0\le l\le L} \left[ u(c,l)-(c+wl)y\right] , \end{aligned}$$

which will help us to reduce the number of control variables up to a single one.

#### Lemma 2.1

The convex dual transform of the utility function *u*(*c*, *l*) is

$$\begin{aligned} \tilde{u}(y)=\left[ A_{1}y^{\frac{\delta (1-k)}{\delta (1-k)-1}}-w L y\right] \mathbb {I}_{\{0<y<\tilde{y}\}}+\left[ A_{2}y^{-\frac{1-k}{k}}\right] \mathbb {I}_{\{y\ge \tilde{y}\}}, \end{aligned}$$

with

$$\begin{aligned} A_{1}\triangleq \frac{1\!-\!\delta \!+\!\delta k}{\delta (1\!-\!k)}L^{\!-\!\frac{(1\!-\!k)(1\!-\!\delta )}{\delta (1\!-\!k)\!-\!1}},\quad A_{2}\triangleq \frac{k}{\delta (1\!-\!k)}\left( \frac{1\!-\!\delta }{\delta w}\right) ^{\frac{(1\!-\!k)(1\!-\!\delta )}{k}}, \quad \text{ and }\quad \tilde{y}\triangleq L^{\!-k}\left( \frac{1\!-\!\delta }{\delta w}\right) ^{1\!-\!\delta (1\!-\!k)}. \end{aligned}$$

Furthermore, the consumption-leisure policy reaching the supremum in (2.4) is

$$\begin{aligned} {\left\{ \begin{array}{ll} \hat{c}=y^{-\frac{1}{k}}\left( \frac{1-\delta }{\delta w}\right) ^{\frac{(1-k)(1-\delta )}{k}}\mathbb {I}_{\{y\ge \tilde{y}\}}+L^{-\frac{(1-k)(1-\delta )}{\delta (1-k)-1}}y^{\frac{1}{\delta (1-k)-1}}\mathbb {I}_{\{0<y<\tilde{y}\}},\\ \hat{l}=y^{-\frac{1}{k}}\left( \frac{1-\delta }{\delta w}\right) ^{-\frac{\delta (1-k)-1}{k}}\mathbb {I}_{\{y\ge \tilde{y}\}}+L\mathbb {I}_{\{0<y<\tilde{y}\}}. \end{array}\right. } \end{aligned}$$

#### Proof

See Appendix A.1. $$\square $$

In this framework, the primal optimization problem, which is denoted as (*P*), is expressed as 
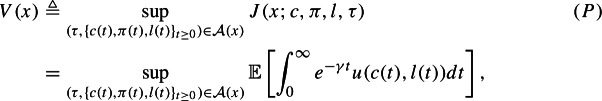
$\mathcal {A}(x)$ stands for the admissible control set and follows the definition below.

#### Remark 2.1

The above framework is consistent with an infinitely lived agent or an agent which death is modelled as the first jump time of an independent Poisson process. In the first case, $\gamma$ is the subjective discount rate. In the second case, we have $\gamma =\hat{\gamma }+\lambda _{D}$, where $\hat{\gamma }$ is the subjective discount rate and $\lambda _{D}$ is the intensity of the Poisson process. In fact, if $\tau _{D}$ is the time in which the death occurs, we have

$$\begin{aligned} \mathbb {E} \left[ \int _{0}^{\tau _D{}}e^{-\hat{\gamma } t}u(c(t),l(t))dt\right]= & {} \mathbb {E} \left[ \int _{0}^{+\infty }e^{-\hat{\gamma } t}e^{-{\lambda _{D}} t}u(c(t),l(t))dt\right] \\= & {} \mathbb {E} \left[ \int _{0}^{+\infty }e^{-{\gamma } t}u(c(t),l(t))dt\right] , \end{aligned}$$

due to the independence of the Poisson process.

#### Definition 2.1

Given the initial wealth $x\ge F+\eta$, $\mathcal {A}(x)$ is defined as the set of all admissible policies satisfying:

- $\tau \le \infty$ is an $\mathbb {F}$-stopping time,
- $\{c(t)\!:\!t\!\ge \! 0\}$ is an $\mathbb {F}$-progressively measurable and non-negative process such that $\int _{0}^{t}\!c(s)ds\!<\!\infty$, a.s., $\forall t\ge 0$,
- $\{\pi (t):t\ge 0\}$ is an $\mathbb {F}$-progressively measurable process such that $\int _{0}^{t}\pi ^{2}(s)ds<\infty$, a.s., $\forall t\ge 0$,
- $\{l(t):t\ge 0\}$ is an $\mathbb {F}$-progressively measurable and non-negative process such that $0\le l(t)\le L$, $\forall t\ge 0$,
- $X(t)\ge F+\eta$ for $0\le t\le \tau$, and $X(t)\ge 0$ for $t>\tau$ a.s.,
- $$\mathbb {E}\left[ \int _{0}^{\infty }e^{-\gamma t}u^{-}(c(t),l(t))dt\right] <\infty $$ with $$u^{-}\triangleq -\min (u,0)$$.

Moreover, we assume $F+\eta \ge \frac{d-w\bar{L}}{r}$, since $\frac{d-w\bar{L}}{r}$ represents the market value of the debt repayment reduced by the maximum amount to borrow against the future income in the pre-bankruptcy period: the agent is therefore unable to allocate the investment and consumption when the wealth level stays below it.

The subsequent proposition provides the corresponding budget constraint.

#### Proposition 2.1

Given any initial wealth $x\ge F+\eta$, any strategy $\left( \tau ,\{c(t),\pi (t),l(t)\}_{t\ge 0}\right) \in \mathcal {A}(x)$, the budget constraint is given by

2.5 $$\begin{aligned} \mathbb {E}\left[ \int _{0}^{\tau }H(t)\left( c(t)+d+wl(t)-w\bar{L}\right) dt+H(\tau )X(\tau )\right] \le x. \end{aligned}$$

#### Proof

See Appendix A.2. $$\square $$

Completing the construction of the primal optimization problem, we are going to solve it explicitly in the following sections. The gain function of the primal optimization problem (*P*) can be rewritten as the expectation of two separated terms representing the pre- and post-bankruptcy part,

$$\begin{aligned} \begin{aligned} J(x;c,\pi ,l,\tau )&=\mathbb {E} \left[ \int _{0}^{\infty }e^{-\gamma t}u(c(t),l(t))dt\right] \\&=\mathbb {E} \left[ \int _{0}^{\tau }e^{-\gamma t}u(c(t),l(t))dt+e^{-\gamma \tau }\mathbb {E}\left[ \left. \int _{\tau }^{\infty }e^{-\gamma (t-\tau )}u(c(t),l(t))dt\right| \mathcal {F}_{\tau }\right] \right] \\&=\mathbb {E} \left[ \int _{0}^{\tau }e^{-\gamma t}u(c(t),l(t))dt+e^{-\gamma \tau }J_{\scriptscriptstyle PB}(\alpha (X(\tau )-F);c,\pi ,l)\right] , \end{aligned} \end{aligned}$$

where we define $J_{\scriptscriptstyle PB}(X(t);c,\pi ,l)\triangleq \mathbb {E} \left[ \left. \int _{t}^{\infty }e^{-\gamma (s-t)}u(c(s),l(s))ds\right| \mathcal {F}_{t}\right]$ in the post-bankruptcy framework, i.e., no debt repayment. We perform the backward approach, hence, begin with the post-bankruptcy part by means of the dynamic programming principle.

## Post-bankruptcy problem

We first tackle the post-bankruptcy problem, assuming without loss of generalization $\tau =0$. Since the debt repayment is removed from the wealth process after the bankruptcy, the corresponding dynamics becomes

$$\begin{aligned} {\left\{ \begin{array}{ll} d X(t)=[rX(t)+\pi (t)(\mu -r)-c(t)+w(\bar{L}-l(t))]dt+\sigma \pi (t)dB(t),\\ X(0)=x. \end{array}\right. } \end{aligned}$$

Afterwards, based on the gain function $J_{\scriptscriptstyle PB}(\cdot )$ defined at the end of the previous section, we can express the value function of the post-bankruptcy part as follows, 
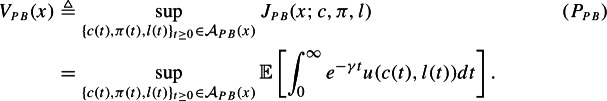
 The admissible control set $\mathcal {A}_{\scriptscriptstyle PB}(x)$ is compatible with Definition 2.1, only removing the condition about the stopping time and changing the liquidity condition from “$X(t)\ge F+\eta$ for $0\le t\le \tau$, and $X(t)\ge 0$ for $t>\tau$ a.s.” to “$X(t)\ge 0$ for $t\ge 0$ a.s.”. Additionally, for any given initial endowment $x\ge 0$ and admissible consumption-portfolio-leisure strategy $\{c(t),\pi (t),l(t)\}_{\scriptscriptstyle t\ge 0}\in \mathcal {A}_{\scriptscriptstyle PB}(x)$, the following propositions provide us the budget and liquidity constraint to the post-bankruptcy problem.

### Proposition 3.1

The infinite horizon budget constraint of the post-bankruptcy problem is

$$\begin{aligned} \mathbb {E}\left[ \int _{0}^{\infty }H(t)(c(t)+wl(t)-w\bar{L})dt\right] \le x. \end{aligned}$$

### Proof

Similarly as in Appendix A.2, we can prove that $\mathbb {E}\left[ \int _{0}^{t}H(s)\left( c(s)+wl(s)-w\bar{L}\right) ds\right] \le x$. Then the above budget constraint can be obtained by taking the limit as $t\rightarrow \infty$. $\square$

### Proposition 3.2

The infinite horizon liquidity constraint of the post-bankruptcy problem is

$$\begin{aligned} \mathbb {E}\left[ \left. \int _{t}^{\infty }\frac{H(s)}{H(t)}(c(s)+wl(s)-w\bar{L})ds\right| \mathcal {F}_{t}\right] \ge 0. \end{aligned}$$

### Proof

The result directly comes from Karatzas and Shreve (1998, Section 3.9, Theorem 9.4), more precisely, the non-negative property of *X*(*t*) and $\mathbb {E}\Big [\left. \int _{t}^{\infty }\frac{H(s)}{H(t)}(c(s)+wl(s)-w\bar{L})ds\right| \mathcal {F}_{t}\Big ]=X(t)$. $\square$

Then, we implement the methodology presented in Karatzas and Wang (2000) and He and Pagès (1993) to establish a duality between the optimal control problem and the individual’s shadow price problem through the Lagrange method. We make the following derivation of $J_{\scriptscriptstyle PB}(x;c,\pi ,l)$, introducing a non-increasing process $D_{\scriptscriptstyle PB}(t)\!\ge \! 0$ and a Lagrange multiplier $\lambda$,

$$\begin{aligned} \begin{aligned} J_{\scriptscriptstyle PB}(x;c,\pi ,l)&=\mathbb {E}\left[ \int _{0}^{\infty }e^{-\gamma t}\left( u(c(t),l(t))-(c(t)+wl(t))\lambda e^{\gamma t}D_{\scriptscriptstyle PB}(t)H(t) \right) dt\right] \\&\qquad +\lambda \mathbb {E}\left[ \int _{0}^{\infty }(c(t)+wl(t))D_{\scriptscriptstyle PB}(t)H(t)dt\right] \\&\le \mathbb {E} \left[ \int _{0}^{\infty }e^{-\gamma t}\tilde{u}(\lambda e^{\gamma t}D_{\scriptscriptstyle PB}(t)H(t))dt\right] +\lambda \mathbb {E}\left[ \int _{0}^{\infty }(c(t)+wl(t))D_{\scriptscriptstyle PB}(t)H(t)dt\right] \\&=\mathbb {E} \left[ \int _{0}^{\infty }e^{-\gamma t}\left( \tilde{u}(\lambda e^{\gamma t}D_{\scriptscriptstyle PB}(t)H(t))+w\bar{L}\lambda e^{\gamma t}D_{\scriptscriptstyle PB}(t)H(t)\right) dt\right] \\&\qquad +\lambda \mathbb {E}\left[ \int _{0}^{\infty }(c(t)+wl(t)-w\bar{L})D_{\scriptscriptstyle PB}(t)H(t)dt\right] . \end{aligned} \end{aligned}$$

By the Fubini’s Theorem, see e.g. Björk (2009, Appendix A, Theorem A.48), we have

$$\begin{aligned} \begin{aligned} \int _{0}^{\infty }H(t)D_{\scriptscriptstyle PB}(t)(c(t)+wl(t)-w\bar{L})dt&= \int _{0}^{\infty }H(t)(c(t)+wl(t)-w\bar{L})dt\\&\quad +\!\int _{0}^{\infty }\!H(t)\!\int _{t}^{\infty }\!\frac{H(s)}{H(t)}(c(s)\!+\!wl(s)\!-\!w\bar{L})dsdD_{\scriptscriptstyle PB}(t), \end{aligned} \end{aligned}$$

and the inequality concerning $J_{\scriptscriptstyle PB}(x;c,\pi ,l)$ can be rewritten as

$$\begin{aligned} \begin{aligned} J_{\scriptscriptstyle PB}(x;c,\pi ,l)&\le \mathbb {E}\left[ \int _{0}^{\infty }e^{-\gamma t}\left( \tilde{u}(\lambda e^{\gamma t}D_{\scriptscriptstyle PB}(t)H(t))+w\bar{L}\lambda e^{\gamma t}D_{\scriptscriptstyle PB}(t)H(t)\right) dt\right] \\&\qquad +\lambda \mathbb {E}\left[ \int _{0}^{\infty }H(t)(c(t)+wl(t)-w\bar{L})dt\right] \\&\qquad +\lambda \mathbb {E}\left[ \int _{0}^{\infty }H(t)\mathbb {E}\left[ \left. \int _{t}^{\infty }\frac{H(s)}{H(t)}(c(s)+wl(s)-w\bar{L})ds\right| \mathcal {F}_{t}\right] dD_{\scriptscriptstyle PB}(t)\right] \\&\le \mathbb {E}\left[ \int _{0}^{\infty }e^{-\gamma t}\left( \tilde{u}(\lambda e^{\gamma t}D_{\scriptscriptstyle PB}(t)H(t))+w\bar{L}\lambda e^{\gamma t}D_{\scriptscriptstyle PB}(t)H(t)\right) dt\right] +\lambda x, \end{aligned} \end{aligned}$$

the last inequality is derived from the budget constraint, the liquidity constraint and the non-increasing property of $D_{\scriptscriptstyle PB}(t)$. Referring to He and Pagès (1993, Section 4), we can define the corresponding individual’s shadow price problem $(S_{\scriptscriptstyle PB})$ as below: 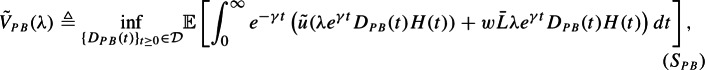


where $\mathcal {D}$ represents the set of non-negative, non-increasing and progressively measurable processes. Then, we construct the duality between the optimal consumption-portfolio-leisure problem $(P_{\scriptscriptstyle PB})$ and the individual’s shadow price problem $(S_{\scriptscriptstyle PB})$.

### Theorem 3.1

(Duality theorem) Suppose $D_{\scriptscriptstyle PB}^{*}(t)$ is the optimal solution (the $\text {arg\,inf}$) to the dual shadow price problem $(S_{\scriptscriptstyle PB})$, then the optimal consumption-leisure strategy to the primal problem $(P_{\scriptscriptstyle PB})$ satisfies $c^{*}(t)+wl^{*}(t)=-\tilde{u}^{\prime }(\lambda ^* e^{\gamma t}D_{\scriptscriptstyle PB}^{*}(t)H(t))$, and we have the following relation

$$\begin{aligned} V_{\scriptscriptstyle PB}(x)=\inf _{\lambda >0} \left\{ \tilde{V}_{\scriptscriptstyle PB}(\lambda )+\lambda x\right\} , \qquad \forall x\ge 0. \end{aligned}$$

Here $\lambda ^*$ is the parameter $\lambda$ which gives the infimum in the above equation.

### Proof

See Appendix B.1. $$\square $$

The above duality theorem states that the optimal solution of the post-bankruptcy problem is transformed into finding the optimal $D_{\scriptscriptstyle PB}^{*} (t)$. In order to solve the problem $(S_{\scriptscriptstyle PB})$ explicitly, we follow the approach in Davis and Norman (1990) and first provide the subsequent assumption.

### Assumption 3.1

The non-increasing process $D_{\scriptscriptstyle PB}(t)$ is absolutely continuous with respect to *t*. Hence, there exists a process $\psi _{\scriptscriptstyle PB}(t)\ge 0$ such that $dD_{\scriptscriptstyle PB}(t)=-\psi _{\scriptscriptstyle PB}(t)D_{\scriptscriptstyle PB}(t)dt$.

Defining a new process $Z(t) \triangleq \lambda e^{\gamma t} D_{\scriptscriptstyle PB}(t)H(t)$, the value function of problem $(S_{\scriptscriptstyle PB})$ can be rewritten as $\tilde{V}_{\scriptscriptstyle PB}(\lambda )=\inf \limits _{\psi _{\scriptscriptstyle PB}(t)\ge 0} \mathbb {E}\left[ \int _{0}^{\infty }e^{-\gamma t}(\tilde{u}(Z(t))+w\bar{L}Z(t))dt\right]$, where *Z*(*t*) is the state variable, and follows the dynamics

3.1 $$\begin{aligned} {\left\{ \begin{array}{ll} \frac{dZ(t)}{Z(t)}=\frac{dD_{\scriptscriptstyle PB}(t)}{D_{\scriptscriptstyle PB}(t)}+(\gamma -r)dt-\theta dB(t)=-\psi _{\scriptscriptstyle PB}(t)dt+(\gamma -r)dt-\theta dB(t),\\ Z(0)=\lambda >0. \end{array}\right. } \end{aligned}$$

Then we introduce a new function $\phi _{\scriptscriptstyle PB}:(\mathbb {R}^{+},\mathbb {R}^{+})\mapsto \mathbb {R}$ as

3.2 $$\begin{aligned} \phi _{\scriptscriptstyle PB}(t,z)\triangleq \inf _{\psi _{\scriptscriptstyle PB}(t)\ge 0} \mathbb {E}\left[ \left. \int _{t}^{\infty } e^{-\gamma s} \left( \tilde{u}(Z(s))+w\bar{L}Z(s)\right) ds\right| Z(t)=z\right] , \end{aligned}$$

which implies that $\tilde{V}_{\scriptscriptstyle PB}(\lambda )=\phi _{\scriptscriptstyle PB}(0,\lambda )$. The Bellman equation associated to $\phi _{\scriptscriptstyle PB}(t,z)$ is

3.3 $$\begin{aligned} \min _{\psi _{\scriptscriptstyle PB}\ge 0}\left\{ \tilde{\mathcal {L}}\phi _{\scriptscriptstyle PB}(t,z)+e^{-\gamma t}(\tilde{u}(z)+w\bar{L}z)-\gamma \phi _{\scriptscriptstyle PB}(t,z)-\psi _{\scriptscriptstyle PB} z\frac{\partial \phi _{\scriptscriptstyle PB}}{\partial z}(t,z) \right\} =0, \end{aligned}$$

with the operator $\tilde{\mathcal {L}}=(\gamma -r)z\frac{\partial }{\partial z}+\frac{1}{2}\theta ^{2}z^{2}\frac{\partial ^{2}}{\partial z^{2}}$. The above Bellman equation implies the following characterizations for the optimum $\psi _{\scriptscriptstyle PB}^{*}$ (the arg min):

$$\begin{aligned} \frac{\partial \phi _{\scriptscriptstyle PB}}{\partial z}(t,z)=0 \Rightarrow \psi _{\scriptscriptstyle PB}^{*}\ge 0;\qquad \frac{\partial \phi _{\scriptscriptstyle PB}}{\partial z}(t,z)\le 0 \Rightarrow \psi _{\scriptscriptstyle PB}^{*}= 0. \end{aligned}$$

Moreover, the Bellman Equation (3.3) can be transformed into

$$\begin{aligned} \min \left\{ \tilde{\mathcal {L}}\phi _{\scriptscriptstyle PB}(t,z)-\gamma \phi _{\scriptscriptstyle PB}(t,z)+e^{-\gamma t}(\tilde{u}(z)+w\bar{L}z),-\frac{\partial \phi _{\scriptscriptstyle PB}}{\partial z}(t,z)\right\} =0, \end{aligned}$$

which corresponds to the variational inequalities: find a function ${\phi }_{\scriptscriptstyle PB}(\cdot ,\cdot )\in C^{2}((0,\infty )\times \mathbb {R}^{+})$ and a free-boundary $\hat{z}_{PB}$, satisfying

3.4 $$\begin{aligned} {\left\{ \begin{array}{ll} (V1) \quad \frac{\partial {\phi }_{\scriptscriptstyle PB}}{\partial z}(t,z)=0, &{} z\ge \hat{z}_{\scriptscriptstyle PB},\\ (V2) \quad \frac{\partial {\phi }_{\scriptscriptstyle PB}}{\partial z}(t,z)\le 0, &{} 0<z<\hat{z}_{\scriptscriptstyle PB},\\ (V3)\quad \tilde{\mathcal {L}}{\phi }_{\scriptscriptstyle PB}(t,z)-\gamma {\phi }_{\scriptscriptstyle PB}(t,z)+e^{-\gamma t}(\tilde{u}(z)+w\bar{L}z)=0, &{} 0<z<\hat{z}_{\scriptscriptstyle PB},\\ (V4)\quad \tilde{\mathcal {L}}{\phi }_{\scriptscriptstyle PB}(t,z)-\gamma {\phi }_{\scriptscriptstyle PB}(t,z)+e^{-\gamma t}(\tilde{u}(z)+w\bar{L}z)\ge 0, &{} z\ge \hat{z}_{\scriptscriptstyle PB}, \end{array}\right. } \end{aligned}$$

for any $t\ge 0$, with the smooth fit conditions $\frac{\partial {\phi }_{\scriptscriptstyle PB}}{\partial z}(t,\hat{z}_{\scriptscriptstyle PB})=0$, $\frac{\partial ^{2}{\phi }_{\scriptscriptstyle PB}}{\partial z^{2}}(t,\hat{z}_{\scriptscriptstyle PB})=0$. In line with Choi et al. (2008, Appendix A), we assume that ${\phi }_{\scriptscriptstyle PB}(t,z)$ takes the time-independent form ${\phi }_{\scriptscriptstyle PB}(t,z)=e^{-\gamma t}v_{\scriptscriptstyle PB}(z)$ to solve the above variational inequalities explicitly. The solution is computed in Appendix B.2 in a semi-analytical framework, i.e., as the solution of a non-linear system of equations. Once the function ${\phi }_{\scriptscriptstyle PB}(\cdot ,\cdot )$ is computed, and therefore $\tilde{V}_{\scriptscriptstyle PB}(\cdot )$ is known, we can recover ${V}_{\scriptscriptstyle PB}(\cdot )$ from Theorem 3.1.

## Primal optimization problem

Once we solved the post-bankruptcy problem, we first of all have to deal with the jump in the wealth at bankruptcy time. We introduce a subset of the primal optimization problem’s admissible control set, $\mathcal {A}_{1}(x)\subset \mathcal {A}(x)$, inside which any policy maximises the gain function of the post-bankruptcy problem. That is to say, for any $(\tau , \{c(t),\pi (t),l(t) \})\in \mathcal {A}_{1}(x)$, it holds:

$$\begin{aligned} \mathbb {E}\left[ \int _{\tau }^{\infty }e^{-\gamma t}u(c(t),l(t))dt\right] =\mathbb {E}\left[ e^{-\gamma \tau }V_{\scriptscriptstyle PB}(\alpha (X^{x,c,\pi ,l}(\tau )-F))\mathbb {I}_{\{\tau <\infty \}}\right] . \end{aligned}$$

Here $X^{x,c,\pi ,l}(\tau )$ is the wealth at time $\tau$ given an initial wealth *x* and assuming the policies $c,\pi ,l$ for consumption, allocation in the risky asset and leisure rate, respectively. Then, from the dynamic programming principle, the whole optimization problem is converted into

$$\begin{aligned} V(x)\triangleq \sup _{(\tau , \{c(t),\pi (t),l(t) \}_{\scriptscriptstyle t\ge 0})\in \mathcal {A}_{1}(x)} \mathbb {E}\left[ \int _{0}^{\tau }e^{-\gamma t}u(c(t),l(t))dt+e^{-\gamma \tau }U(X^{x,c,\pi ,l}(\tau ))\right] , \end{aligned}$$

denoting the value function at the moment of bankruptcy as

$$\begin{aligned} U(X^{x,c,\pi ,l}(\tau ))\triangleq \sup _{\{c(t),\pi (t),l(t) \}_{\scriptscriptstyle t\ge 0}\in \mathcal {A}_{1}(x)} \mathbb {E}\left[ \left. \int _{\tau }^{\infty }e^{-\gamma (s-\tau )}u(c(s),l(s))ds\right| \mathcal {F}_{\tau }\right] . \end{aligned}$$

Therefore, it can be observed that the relationship between $U(\cdot )$ and the post-bankruptcy value function $V_{\scriptscriptstyle PB}(\cdot )$ is

$$\begin{aligned} U(X^{x,c,\pi ,l}(\tau ))=V_{\scriptscriptstyle PB}(\alpha (X^{x,c,\pi ,l}(\tau )-F)). \end{aligned}$$

Simple computations give us the Legendre–Fenchel transform of *U*(*x*), $\tilde{U}(z)$, that is

4.1 $$\begin{aligned} \tilde{U}(z)=\tilde{V}_{\scriptscriptstyle PB}\left( \frac{z}{\alpha }\right) -Fz={\phi }_{\scriptscriptstyle PB}\left( 0,\frac{z}{\alpha }\right) -Fz. \end{aligned}$$

Obtained the optimal solution for the post-bankruptcy problem, we now reduce the primal optimization problem by fixing the stopping time. Defining a set of admissible controls corresponding to a fixed stopping time $\tau \in \mathcal {T}$ as

$$\begin{aligned} \mathcal {A}_{\tau }(x)\triangleq \left\{ \{c(t),\pi (t),l(t)\}_{t\ge 0}: \left( \tau ,\{c(t),\pi (t),l(t)\}_{t\ge 0}\right) \in \mathcal {A}(x) \,\text{ for } \text{ any } \text{ fixed } \, \tau \in \mathcal {T} \right\} , \end{aligned}$$

and a utility maximization problem as 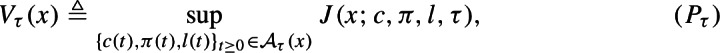


the problem (*P*) can be transformed into an optimal stopping time problem, $V(x)=\sup \limits _{\tau \in \mathcal {T}}V_{\tau }(x)$. Then, we put forward the liquidity constraint for the optimal bankruptcy problem.

### Proposition 4.1

The liquidity constraint of the considered problem is

4.2 $$\begin{aligned} \mathbb {E}\left[ \left. \int _{t}^{\tau }\frac{H(s)}{H(t)}\left( c(s)+d+wl(s)-w\bar{L}\right) ds+\frac{H(\tau )}{H(t)}X(\tau )\right| \mathcal {F}_{t}\right] \ge F+\eta , \forall t\in [0,\tau ].\nonumber \\ \end{aligned}$$

### Proof

See Appendix C.1. $$\square $$

Following the same technique as in the post-bankruptcy problem, considering the budget and liquidity constraints (2.5) and (4.2), we introduce a real number $\lambda > 0$, the Lagrange multiplier, and a non-increasing continuous process $D(t)>0$. We obtain

$$\begin{aligned} \begin{aligned} J(x;c,\pi ,l,\tau )&\le \mathbb {E}\bigg [\int _{0}^{\tau }e^{-\gamma t}\left( \tilde{u}(\lambda D(t)e^{\gamma t}H(t))-(d-w\bar{L})\lambda e^{\gamma t}D(t)H(t)\right) dt\\&\qquad +e^{-\gamma \tau }\tilde{U}(\lambda D(\tau )e^{\gamma \tau }H(\tau ))\bigg ] +\lambda \mathbb {E}\left[ \int _{0}^{\tau }(F+\eta )H(t)dD(t)\right] +\lambda x. \end{aligned} \end{aligned}$$

As in He and Pagès (1993, Section 4), the individual’s shadow price problem inspired by the above inequality is defined as 
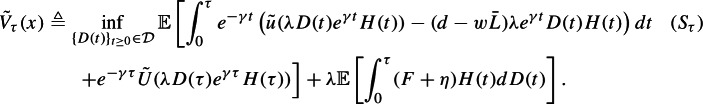


Hereafter, we provide a theorem to construct the duality between the individual’s shadow price problem $(S_{\tau })$ and the optimal consumption-portfolio-leisure problem $(P_{\tau })$.

### Theorem 4.1

(Duality theorem) Suppose $D^{*}(t)$ is the optimal solution (the $\text {arg\,inf}$) to Problem $(S_{\tau })$, then $c^{*}(t)+wl^{*}(t)=-\tilde{u}^{\prime }(\lambda ^* e^{\gamma t}D^{*}(t)H(t))$ and $X^{x,c^{*},\pi ^{*},l^{*}}(\tau )=-\tilde{U}^{\prime }(\lambda ^* e^{\gamma \tau }D^{*}(\tau )H(\tau ))$ coincide with the optimal solution to Problem $(P_{\tau })$, and we have the following relationship

$$\begin{aligned} V_{\tau }(x)=\inf _{\lambda >0} \left\{ \tilde{V}_{\tau }(\lambda )+\lambda x\right\} , \qquad \forall x\ge F+\eta . \end{aligned}$$

Here $\lambda ^*$ is the parameter $\lambda$ which gives the infimum in the above equation.

### Proof

See Appendix C.2. $$\square $$

Furthermore, the duality theorem gives the value function of problem (*P*) as:

$$\begin{aligned} V(x)=\sup _{\tau \in \mathcal {T}}V_{\tau }(x)=\sup _{\tau \in \mathcal {T}}\inf _{\lambda>0}\{\tilde{V}_{\tau }(\lambda )+\lambda x\}\le \inf _{\lambda>0}\sup _{\tau \in \mathcal {T}}\{\tilde{V}_{\tau }(\lambda )+\lambda x\}=\inf _{\lambda >0}\{\sup _{\tau \in \mathcal {T}}\tilde{V}_{\tau }(\lambda )+\lambda x\}. \end{aligned}$$

Let us introduce a new function $\tilde{V}(\lambda )\triangleq \sup \limits _{\tau \in \mathcal {T}}\tilde{V}_{\tau }(\lambda )$. As in Karatzas and Wang (2000, Section 8, Theorem 8.5), the value function *V*(*x*) satisfies $V(x)=\inf \limits _{\lambda >0} [\tilde{V}(\lambda )+\lambda x]$ under the condition that $\tilde{V}(\lambda )$ exists and is differentiable for any $\lambda > 0$. Hence, the objective optimization problem consists of two steps:

$$\begin{aligned} {\left\{ \begin{array}{ll} \tilde{V}(\lambda )=\sup \limits _{\tau \in \mathcal {T}}\tilde{V}_{\tau }(\lambda ),\\ V(x)=\inf \limits _{\lambda >0} [\tilde{V}(\lambda )+\lambda x]=\tilde{V}(\lambda ^{*})+\lambda ^{*} x. \end{array}\right. } \end{aligned}$$

The first step involves an optimal stopping time problem, while the second refers to obtain the optimum $\lambda ^{*}$ achieving the infimum part. We begin with the first optimization problem related to the individual’s shadow price. Before this, following the method of Davis and Norman (1990), an assumption is imposed on the process *D*(*t*).

### Assumption 4.1

The non-increasing process *D*(*t*) is absolutely continuous with respect to *t*. Hence, there is a non-negative process $\psi (t)$ such that $dD(t)=-\psi (t)D(t)dt$.

Introducing a new process $Z(t)\triangleq \lambda D(t)e^{\gamma t}H(t)$, the value function of the dual problem $(S_{\tau })$ is rewritten as

$$\begin{aligned} \tilde{V}_{\tau }(\lambda )=\inf _{\psi (t)\ge 0}\mathbb {E}\left[ \int _{0}^{\tau } e^{-\gamma t} \left( \tilde{u}(Z(t))-(d-w\bar{L})Z(t)-(F+\eta )\psi (t)Z(t)\right) dt+e^{-\gamma \tau }\tilde{U}(Z(\tau ))\right] . \end{aligned}$$

We consider a new generalized optimization problem

$$\begin{aligned} \phi (t,\!z)\!\triangleq \!\sup _{\tau \ge t} \!\inf _{\psi (t)>0}\! \mathbb {E}\!\left[ \!\left. \int _{t}^{\tau }\!\!\!\!\!e^{\!-\!\gamma s}\! \left( \tilde{u}(Z(s))\!-\!(d\!-\!w\bar{L})Z(s)\!-\!(F\!+\!\eta )\psi (s)Z(s)\right) ds\!+\!e^{\!-\!\gamma \tau }\!\tilde{U}(Z(\tau ))\right| \!Z(t)\!=\!z\right] , \end{aligned}$$

it can be observed that $\tilde{V}(\lambda )=\phi (0,\lambda )$, which indicates the solution of $\tilde{V}(\lambda )$ is resorted to solve the above generalized problem. We start with the infimum part through defining

$$\begin{aligned} \phi _{\scriptscriptstyle inf}(t,z)\!\triangleq \!\!\!\inf _{\psi (t)>0} \!\!\mathbb {E}\!\left[ \left. \int _{t}^{\tau } \!e^{-\gamma s}\! \left( \tilde{u}(Z(s))\!\!-\!\!(d\!-\!w\bar{L})Z(s)\!\!-\!\!(F\!+\!\eta )\psi (s)Z(s)\right) \!ds\!+\!e^{-\gamma \tau }\!\tilde{U}(Z(\tau ))\right| \!Z(t)\!=\!z\right] , \end{aligned}$$

the dynamic programming principle gives us the subsequent Bellman equation

$$\begin{aligned} \min _{\psi \ge 0} \left\{ \mathcal {L}\phi _{\scriptscriptstyle inf}(t,z)+e^{-\gamma t}\left( \tilde{u}(z)-(d-w\bar{L})z\right) -\psi z\left[ \frac{\partial \phi _{\scriptscriptstyle inf}}{\partial z}(t,z)+(F+\eta )e^{-\gamma t}\right] \right\} =0, \end{aligned}$$

being the operator $\mathcal {L}:=\frac{\partial }{\partial t}+(\gamma -r)z\frac{\partial }{\partial z}+\frac{1}{2}\theta ^{2}z^{2}\frac{\partial ^{2}}{\partial z^{2}}$. The following characterizations hold for the optimum $\psi ^{*}$ (the $\text {arg\,min}$):

- $\frac{\partial \phi _{\scriptscriptstyle inf}}{\partial z}(t,z)+(F+\eta )e^{-\gamma t}=0 \Longrightarrow \psi ^{*}\ge 0$: then we get that there exists a boundary $\hat{z}$ such that
  4.3 $$\begin{aligned} \frac{\partial \phi }{\partial z}(t,z)=\frac{\partial \phi _{\scriptscriptstyle inf}}{\partial z}(t,z)=-(F+\eta )e^{-\gamma t},\quad z\ge \hat{z}. \end{aligned}$$
- $\frac{\partial \phi _{\scriptscriptstyle inf}}{\partial z}(t,z)+(F+\eta )e^{-\gamma t}\le 0 \Longrightarrow \psi ^{*}=0$: in this case, $\phi (t,z)$ is reduced to a pure optimal stopping time problem,
  4.4 $$\begin{aligned} \phi (t,z)\!=\!\sup _{\tau \ge t} \mathbb {E}\left[ \left. \int _{t}^{\tau }\!\!\! e^{\!-\!\gamma s} \left( \tilde{u}(Z(s))\!-\!(d\!-\!w\bar{L})Z(s)\right) ds\!+\!e^{-\gamma \tau }\tilde{U}(Z(\tau ))\right| Z(t)\!=\!z\right] ,\quad 0<z<\hat{z}. \end{aligned}$$

We first focus on the optimal stopping time problem (4.4) and present a lemma to determine its continuous and stopping regions. But before this, one relationship should be noticed. With the optimum $\psi ^{*}(t)$, the function $\phi _{\scriptscriptstyle inf}(t,z)$ can be rewritten as

$$\begin{aligned} \phi _{\scriptscriptstyle inf}(t,z)= \mathbb {E}\left[ \left. \int _{t}^{\tau } \!e^{-\gamma s}\! \left( \tilde{u}(Z(s))\!-\!(d\!-\!w\bar{L})Z(s)\!-\!(F\!+\!\eta )\psi ^{*}(s)Z(s)\right) \!ds\!+\!e^{-\gamma \tau }\!\tilde{U}(Z(\tau ))\right| \!Z(t)\!=\!z\right] . \end{aligned}$$

Fixing the time with $t=\tau$, $\phi _{\scriptscriptstyle inf}(\tau ,z)$ can be treated as a single variable function of *z*, that is,

$$\begin{aligned} \phi _{\scriptscriptstyle inf}(\tau ,z)\!=\!e^{\!-\!\gamma \tau }\tilde{U}(z). \end{aligned}$$

From this equation, and Eqs. (4.1) and (4.3), we obtain $\tilde{U}^{\prime }(\hat{z})=-(F+\eta )\le -F=\tilde{U}^{\prime }(\alpha \hat{z}_{\scriptscriptstyle PB})$; considering the convex property of $\tilde{U}(z)$, we directly obtain the relationship

4.5 $$\begin{aligned} \hat{z}\le \alpha \hat{z}_{\scriptscriptstyle PB}. \end{aligned}$$

### Lemma 4.1

Assuming

4.6 $$\begin{aligned} rF-d+w\bar{L}-\frac{w\bar{L}}{\alpha }<0, \end{aligned}$$

there exists $\bar{z}$ such that the continuous region of the optimal stopping problem (4.4) with the state variable *Z*(*t*) is $\Omega _{1}=\{0<z<\bar{z}\}$, and the stopping region is $\Omega _{2}=\{z\ge \bar{z}\}$. $\bar{z}$ is the boundary that separates $\Omega _{1}$ and $\Omega _{2}$.

### Proof

See Appendix C.3 $$\square $$

**Fig. 1:**
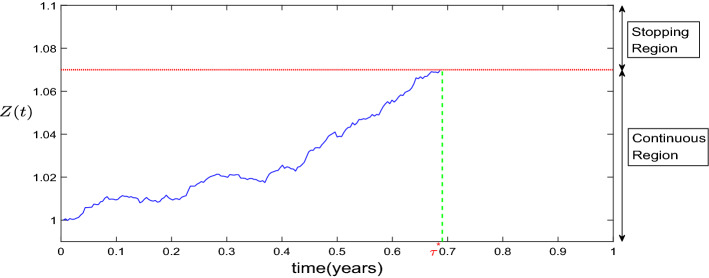
Relationship between optimal bankruptcy time and continuous and stopping regions

After obtaining the continuous region $\Omega _{1}\!=\!\{0\!<\!z\!<\!\bar{z}\}$, we can treat the stopping time of bankruptcy as the first hitting time of process *Z*(*t*) to the boundary $\bar{z}$ from the inner region of $\Omega _{1}$. Figure 1 describes the relationship between the optimal stopping time and the continuous and stopping regions.

The optimal bankruptcy time is the moment when process *Z*(*t*) first touches the boundary, which is represented with the red dotted line, from within the continuous region. Hence, the stopping time satisfies $\tau =\inf \{t\ge 0:Z(t)\ge \bar{z}\}$ and will be proved to be finite with probability one under a sufficient constraint with the following lemma. Besides, it should be clear that $\bar{z}$, corresponding to the bankruptcy threshold, is an upper barrier for the process *Z*(*t*), and therefore a lower barrier of the wealth process, as we will show in Remark 4.1.

### Lemma 4.2

Under the assumption

4.7 $$\begin{aligned} \gamma >r+\frac{\theta ^{2}}{2}, \end{aligned}$$

we have $\mathbb {P}\left( \tau _{\bar{z}}\!<\!\tau _{0}\right) \!=\!1$, with two stopping times, $\tau _{\bar{z}}\!=\!\inf \limits _{t\ge 0}\{t:Z(t)\!=\!\bar{z}\}$ and $\tau _{0}\!=\!\inf \limits _{t\ge 0}\{t:Z(t)\!=\!0\}$.

### Proof

See Appendix C.4. $$\square $$

Subsequently, combining Condition (4.3) and the optimal stopping time problem (4.4), we get the free boundary problem which characterizes the function $\phi (\cdot ,\cdot )$, considering two different cases: (1) *Variational inequalities assuming* $\bar{z}<\hat{z}$: Find the free boundaries $\bar{z}>0$ (Bankruptcy), $\hat{z}>0$ ($(F+\eta )$-wealth level), and a function ${\phi }(\cdot ,\cdot )\in C^{1}((0,\infty )\times \mathbb {R}^{+})\cap C^{2}((0,\infty )\times \mathbb {R}^{+}\setminus \{\bar{z}\})$ satisfying

4.8 $$\begin{aligned} {\left\{ \begin{array}{ll} (V1) \quad \frac{\partial {\phi }}{\partial z}(t,z)+(F+\eta )e^{-\gamma t}=0, &{} z\ge \hat{z},\\ (V2) \quad \frac{\partial {\phi }}{\partial z}(t,z)+(F+\eta )e^{-\gamma t}\le 0, &{} 0<z<\hat{z},\\ (V3)\quad \mathcal {L}{\phi }(t,z)+e^{-\gamma t}\left( \tilde{u}(z)-(d-w\bar{L})z\right) =0, &{} 0<z<\bar{z},\\ (V4)\quad \mathcal {L}{\phi }(t,z)+e^{-\gamma t}\left( \tilde{u}(z)-(d-w\bar{L})z\right) \le 0, &{} \bar{z}\le z<\hat{z},\\ (V5) \quad {\phi }(t,z)\ge e^{-\gamma t}\tilde{U}(z), &{} 0<z<\bar{z},\\ (V6) \quad {\phi }(t,z)= e^{-\gamma t}\tilde{U}(z), &{}\bar{z}\le z<\hat{z}, \end{array}\right. } \end{aligned}$$

for any $t\ge 0$, with the smooth fit conditions

$$\begin{aligned} \frac{\partial {\phi }}{\partial z}(t,\hat{z})= & {} -(F+\eta )e^{-\gamma t},\quad \frac{\partial ^{2}{\phi }}{\partial z^{2}}(t,\hat{z})=0, \\ \frac{\partial {\phi }}{\partial z}(t,\bar{z})= & {} e^{-\gamma t}\tilde{U}^{\prime }(\bar{z}),\quad {\phi }(t,\bar{z})=e^{-\gamma t}\tilde{U}(\bar{z}). \end{aligned}$$

Furthermore, in the period up to the stopping time $\tau$, which corresponds to the interval $0<z<\bar{z}$, we need to consider whether the constraint $0\le l(t)\le L$ is triggered or not, which is related to the boundary $\tilde{y}$ introduced in Lemma 2.1. Hence, the problem of the pre-bankruptcy part is divided into two cases, $0<\tilde{y}\le \bar{z}$ and $0<\bar{z}<\tilde{y}$. Then combining with the two cases of the post-bankruptcy part, we have the following framework of partition for the primal optimization problem (*P*).

- $$0<\bar{z}<\tilde{y}$$: 
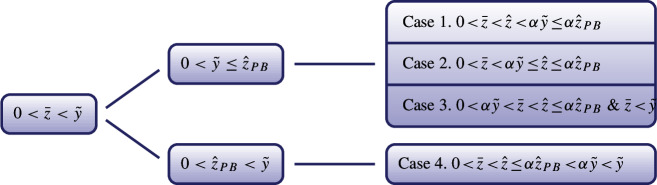
- $$0<\tilde{y}\le \bar{z}$$: 
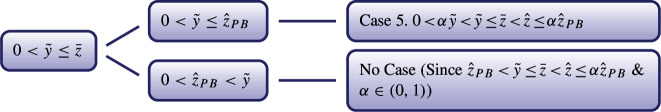

(2) *Variational inequalities assuming* $\bar{z}\ge \hat{z}$: Find the free boundaries $\bar{z}>0$ (Bankruptcy), $\hat{z}>0$ ($(F+\eta )$-wealth level), and a function $\phi (\cdot ,\cdot )\in C^{1}((0,\infty )\times \mathbb {R}^{+})\cap C^{2}((0,\infty )\times \mathbb {R}^{+}\setminus \{\bar{z}\})$ satisfying

4.9 $$\begin{aligned} {\left\{ \begin{array}{ll} (V1) \quad \frac{\partial \phi }{\partial z}(t,z)+(F+\eta )e^{-\gamma t}=0, &{} \hat{z}\le z<\bar{z},\\ (V2) \quad \frac{\partial \phi }{\partial z}(t,z)+(F+\eta )e^{-\gamma t}\le 0, &{} 0<z<\hat{z},\\ (V3)\quad \mathcal {L}\phi (t,z)+e^{-\gamma t}\left( \tilde{u}(z)-(d-w\bar{L})z\right) =0, &{} 0<z<\hat{z},\\ (V4)\quad \mathcal {L}\phi (t,z)+e^{-\gamma t}\left( \tilde{u}(z)-(d-w\bar{L})z\right) \le 0, &{}\hat{z}\le z<\bar{z},\\ (V5) \quad \phi (t,z)\ge e^{-\gamma t}\tilde{U}(z), &{} 0<z<\bar{z},\\ (V6) \quad \phi (t,z)= e^{-\gamma t}\tilde{U}(z), &{}z\ge \bar{z}, \end{array}\right. } \end{aligned}$$

for any $t\ge 0$, with the smooth fit conditions $\frac{\partial \phi }{\partial z}(t,\hat{z})=-(F+\eta )e^{-\gamma t}$ and $\frac{\partial ^{2}\phi }{\partial z^{2}}(t,\hat{z})=0$. Same as before, we provide a diagram to show the possible situations under this prerequisite.
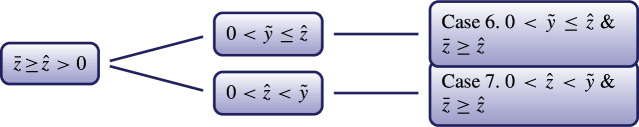


As in the post-bankruptcy problem, we assume that ${\phi }(t,z)$ adopts a time-independent form, ${\phi }(t,z)= e^{-\gamma t} v(z)$, then the solution is obtained, see Appendix C.5. We would like to stress that only one of the seven cases admits a solution.

After acquiring the closed form of *v*(*z*), (Karatzas & Wang, 2000, Section 8, Theorem 8.5) indicates that

$$\begin{aligned} V(x)=\inf \limits _{\lambda>0}[\tilde{V}(\lambda )+\lambda x]=\inf \limits _{\lambda >0}[v(\lambda )+\lambda x] \end{aligned}$$

keeps true for a unique $\lambda ^*>0$ under the differentiable property of $v(\cdot )$. Then the wealth threshold of bankruptcy, namely $\bar{x}$, can be calculated from the relationship $\bar{x}=-v^{\prime }(\bar{z})$. Therefore, given any initial wealth $x\ge F+\eta$, we get the optimal Lagrange multiplier $\lambda ^{*}$ through solving the equation, $x=-v^{\prime } (\lambda ^{*})$. Furthermore, since the optimum $\lambda ^{*}$ is the initial value of process (3.1), $Z^{*}(t)$, the optimal wealth process follows $X^{*}(t)=-v^{\prime }(Z^{*}(t))$. The optimal bankruptcy time satisfies $\tau ^{*}=\inf \limits _{t\ge 0}\{X^{*}(t)\le \bar{x}\}$. Moreover, recalling Lemma 2.1, the optimal consumption and leisure strategies are

$$\begin{aligned} {\left\{ \begin{array}{ll} c^{*}(t)=(Z^{*}(t))^{-\frac{1}{k}}\left( \frac{1-\delta }{\delta w}\right) ^{\frac{(1-k)(1-\delta )}{k}}\mathbb {I}_{\{Z^{*}(t)\ge \tilde{y}\}}+L^{-\frac{(1-k)(1-\delta )}{\delta (1-k)-1}}(Z^{*}(t))^{\frac{1}{\delta (1-k)-1}}\mathbb {I}_{\{0<Z^{*}(t)<\tilde{y}\}},\\ l^{*}(t)=(Z^{*}(t))^{-\frac{1}{k}}\left( \frac{1-\delta }{\delta w}\right) ^{-\frac{\delta (1-k)-1}{k}}\mathbb {I}_{\{Z^{*}(t)\ge \tilde{y}\}}+L\mathbb {I}_{\{0<Z^{*}(t)<\tilde{y}\}}, \end{array}\right. } \end{aligned}$$

and the optimal portfolio strategy is $\pi ^{*}(t)=\frac{\theta }{\sigma }Z^{*}(t)v^{\prime \prime }(Z^{*}(t))$, which can be obtained from He and Pagès (1993, Section 5, Theorem 3).

### Remark 4.1

In Fig. 1, we plot the relationship between the optimal bankruptcy time and the continuous and stopping regions with respect to $Z^*(t)$, showing that the optimal bankruptcy time is the first time the process $Z^*(t)$ touch the upper barrier $\bar{z}$. The same plot can be done with respect to $X^{*}(t)=-v^{\prime }(Z^{*}(t))$: the convex property of $v(\cdot )$, see Karatzas and Shreve (1998, Section 3.4, Lemma 4.3), implies that $X^{*}(t)$ is a decreasing function of $Z^{*}(t)$, therefore, in this case the optimal bankruptcy time is the first time the process $X^*(t)$ touch a lower barrier $\bar{x}=-v^{\prime }(\bar{z})$.

## Numerical analysis

We now implement the sensitivity analysis with respect to the input parameters. As baseline parameters, we consider the ones listed in Table 1. These inputs satisfy conditions (2.3), (4.6) and (4.7).

**Table 1:** Baseline input parameters

$\delta$	*k*	*r*	$\mu$	$\sigma$	$\gamma$	*d*	*w*	*F*	$\eta$	$\alpha$	$\bar{L}$	*L*
0.6	3	0.05	0.1	0.2	0.3	0.3	1.5	0.96	0.0001	0.9	1	0.8

$^\textrm{a}$The definitions of these parameters are summarized in Appendix E

The parameters *r*, $\mu$, $\sigma$, $\gamma$ and $\alpha$ are directly taken from Jeanblanc et al. (2004). Whereas the fixed bankruptcy toll and the debt repayment amount in their study are $F=400\$$ and $d=125\$$, we set $F=0.96$ and $d=0.3$ such that the ratios of *F* and *d* in our and their research keep consistent. As discussed in Sect. 4, seven cases should be considered simultaneously and only one must be verified: in fact with these parameters, only Case 2, “$0<\bar{z}<\alpha \tilde{y}\le \hat{z}<\alpha \hat{z}_{PB}$”, admits a solution. With the above input parameters, we derive the output parameters: $B_{2}$, $\bar{z}$, $\hat{z}$, $\tilde{y}$, the corresponding set of wealth thresholds $\{\hat{x}, \bar{x}, \tilde{x}\}$, the optimal Lagrange multiplier $\lambda ^{*}$ and the value function *V*(*x*). The results are listed in Tables 2 and 3. *fval*, i.e., the value of the function at its zero, in Table 2 represents the maximum error generating from using the *fsolve* function of MATLAB to solve the non-linear equations (C.6), (C.7) and (C.8): as expected, the *fval* value is close to zero, i.e., the algorithm correctly solve the system of equations.

**Table 2:** Output parameters, optimal Lagrange multiplier and value function

$B_{2}$	$\hat{z}$	$\tilde{y}$	$\bar{z}$	$\lambda ^{*}$	*fval*
5.3104	2.6126	0.3280	0.1591	0.3110	3.6973e−11

**Table 3:** Wealth thresholds

$\hat{x}$	$\tilde{x}$		$\bar{x}$
$F+\eta$-wealth level	Minimum wealth level for $l^{*}(t)\!=\!L$		Bankruptcy wealth level
0.9601	6.3164		10.0651

We first of all want to stress that in this case the optimal bankruptcy barrier is $\bar{x}= 10.0651$, i.e., the optimal bankruptcy time is the first time in which the wealth is smaller or equal to 10.0651.

**Fig. 2:**
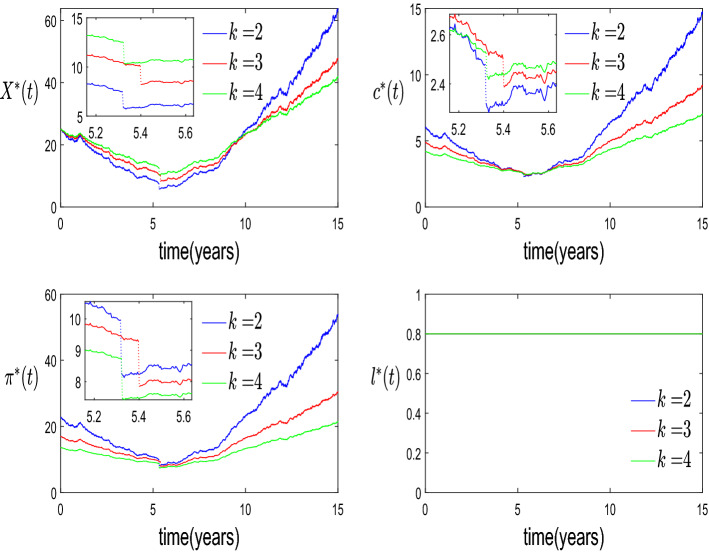
Trajectories of optimal wealth and control strategies w.r.t. risk aversion coefficient

*Sensitivity of optimal solutions with respect to the risk aversion coefficient* *k*: In this part, we use the Monte Carlo method to simulate the single path of optimal wealth process and consumption-portfolio-leisure strategy by taking different values of *k* for discovering the sensitivity of optimal solutions to the risk aversion coefficient. Parameters are the ones in Table 1, with the initial wealth set to $x=25>\bar{x},$ that is, immediate bankruptcy is not optimal. The agent with a higher value of *k* prefers to have a higher wealth threshold for declaring bankruptcy ($\bar{x}=7.4777$ for $k=2$, $\bar{x}=10.0651$ for $k=3$ and $\bar{x}=12.4239$ for $k=4$). This is reasonable, as shown in Fig. 2: the more risk-averse agent tends to smooth the consumption and leisure, and invest less in the risky asset such that maintaining a relatively higher wealth level. Moreover, from the optimal trajectories of leisure, it can be observed that the relatively low fixed and flexible bankruptcy costs, $F = 0.96$ and $1-\alpha = 0.1$, and the high bankruptcy wealth thresholds enable the agent to enjoy the maximum leisure rate $L=0.8$ even after suffering the wealth shrinkage caused by declaring bankruptcy. Therefore, the leisure processes corresponding to different *k* values are fully identical. Numerical results here not reported show that this result is strongly related to the choice of the parameter $\delta$, that is, increasing $\delta$, e.g., $\delta =0.8$, and therefore increasing the utility from consumption and decreasing the one from leisure, the optimal leisure can be lower than *L*. Finally, in Fig. 2 we zoom close to the bankruptcy time, to show the discontinuity of the optimal strategies.

*Sensitivity of the optimal bankruptcy threshold with respect to the market risk premium jointly with the risk aversion coefficient*: The parameter $\theta =\frac{\mu -r}{\sigma }$, which is the Sharpe-Ratio, measures the market risk premium. To discover the relationship between $\bar{x}$ and $\theta$, we keep *r*, $\sigma$ constant and change the value of $\mu$. Table 4 shows that higher is the market risk premium $\theta$ stronger is the ability of the agent to bear the debt repayment; hence, setting a lower wealth threshold to avoid suffering the bankruptcy costs. Furthermore, there is a positive relationship between the optimal wealth threshold of bankruptcy and the risk aversion level *k*.

**Table 4:** Bankruptcy threshold ($\bar{x}$) as a function of the market risk premium and the risk aversion coefficient

	$\theta =0.2$	$\theta =0.4$	$\theta =0.6$	$\theta =0.8$
$k=1.5$	6.3235	5.2612	4.1655	3.2933
$k=2.5$	9.1342	7.6391	6.1001	4.8540

**Table 5:** Bankruptcy threshold ($\bar{x}$) as a function of the flexible bankruptcy cost and the debt repayment

$d=0.1$	$d=0.3$	$d=0.5$	$d=0.7$	$\alpha =0.6$	$\alpha =0.7$	$\alpha =0.8$	$\alpha =0.9$
1.5488	10.0651	20.8392	31.6397	1.2442	1.8441	3.4065	10.0651

*Sensitivity of the optimal bankruptcy threshold with respect to the debt repayment, the fixed and flexible bankruptcy cost*: The wealth process suffers a shrinkage through an affine function $\alpha (X(\tau )-F)$ declaring bankruptcy, and the debt repayment is exempted. Hence, *F* and $(1-\alpha )$ can be treated as the fixed and flexible cost of bankruptcy, and *d* is the benefit of bankruptcy. In order to discover the influence of the bankruptcy option, in Table 5 we observe that $\bar{x}$ is an increasing function of $\alpha$. The rationale is the following: since $\alpha$ represents the proportion of wealth held after bankruptcy, a lower value of $\alpha$ indicates a higher cost such that the agent prefers to set a lower wealth threshold to avoid suffering the wealth shrinkage from bankruptcy. We also have an increasing relationship between $\bar{x}$ and *d*: when the debt repayment is higher, which implies that the benefit of bankruptcy is more attractive, the agent tends to take a higher threshold such that the wealth process satisfies the bankruptcy requirement $\bar{x}$ more easily to enjoy the debt exemption.

**Fig. 3:**
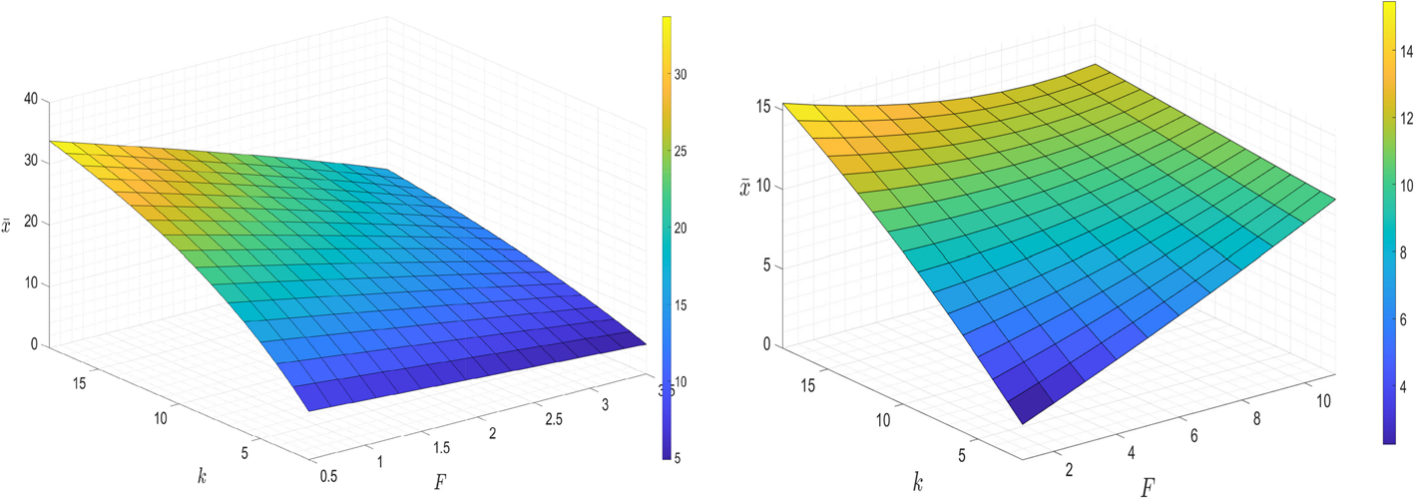
Risk aversion coefficient, fixed bankruptcy cost and bankruptcy wealth threshold

In Fig. 3 we provide three-dimensional images to explain the sensitivity of bankruptcy wealth threshold to the parameter *F* jointly with *k*. Starting from the left figure, since the value of *F* adjusted according to Jeanblanc et al. (2004) is relatively low, the liquidity constraint triggered by $F+\eta$ is easy to be covered by the labour income. Thus, the role of *F* is more related to the fixed cost of bankruptcy such that a higher value of *F* will make the agent set a lower wealth threshold to avoid suffering the bankruptcy, which results in a monotonic decreasing relationship between $\bar{x}$ and *F*. However, *F* is not only the cost of bankruptcy, but also can be regarded as the liquidity constraint to limit the agent’s investment behaviour. In order to reflect the phenomenon that the role of *F* is the trade-off between the liquidity constraint boundary of the pre-bankruptcy period and the fixed cost of bankruptcy, in the right figure we show the same three-dimensional image with different input parameters: in particular, we follow the baseline inputs in Table 1 and only change the values of $r=0.02$, $\mu =0.07$, $\sigma =0.15$, $\gamma =0.1$ and $\alpha =0.7$ to satisfy Condition (4.6) also for larger values of *F*. We find that the relationship between $\bar{x}$ and *F* is not monotonous. When the risk aversion level is low, a positive relationship between *F* and the bankruptcy threshold of wealth is observed. This is because a larger *F*, which is treated as the collateral since $X(t)\ge F+\eta$ before bankruptcy, will reduce the agent’s available capital and limit the investment behaviour. Therefore, the agent will set a higher bankruptcy wealth threshold to get rid of the limitation of the liquidity constraint. Whereas for the agent with deep risk aversion liquidity constraints are less restrictive, and therefore the parameter *F* plays more the role of the bankruptcy cost.

**Fig. 4:**
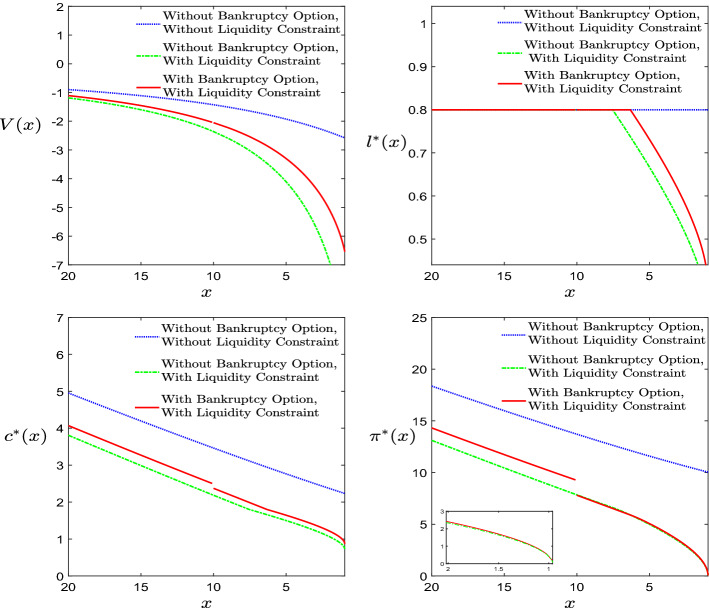
Influence of bankruptcy option

*Influence of the bankruptcy option*: To study the influence of introducing the bankruptcy option, we also solve a pure optimal control problem without optimal stopping, see Appendix D. In Fig. 4, we take the inputs in Table 1 and show the optimal controls (as a function of the initial wealth) for the case with and without bankruptcy option models to reveal its influence. In the second case, we consider both the case with a liquidity constraint, $X(t)>F+\eta$, and without liquidity constraint.

From Fig. 4, five phenomena should be noticed by comparing the two curves with liquidity constraint. First, due to the additional bankruptcy option, the value function is always greater than the value function without such an option at any given initial wealth level. Second, before the occurring of optimal stopping time, the additional option offers the agent a better circumstance, and the optimal consumption-portfolio-leisure policies always dominates the corresponding policies without bankruptcy option model. Third, in order to meet the needs of obtaining utility from consumption and leisure, the agent with the bankruptcy option and a low initial wealth immediately files bankruptcy, facing a shrinkage of wealth, which causes a downward jump in consumption and allocation in the risky asset. Fourth, when a liquidity constraint is considered, the optimal leisure rate decreases for low values of the initial wealth *x*, since the agent needs to spend more time working to get a larger wage, therefore not exploiting the full leisure, to face debt and liquidity constraint. Finally, the amount of money allocated in the risky asset is 0 as the wealth level drops to the liquidity constraint boundary ($F+\eta =0.9601$). This is to avoid that the wealth process violates the liquidity constraint due to the risky asset’s fluctuation. However, the optimal consumption always keeps positive even as the wealth approaches the liquidity boundary since the agent continues to obtain the labour income. Additionally, we can observe that the optimal solutions without the liquidity constraint always dominates the solutions with this extra constraint.

**Fig. 5:**
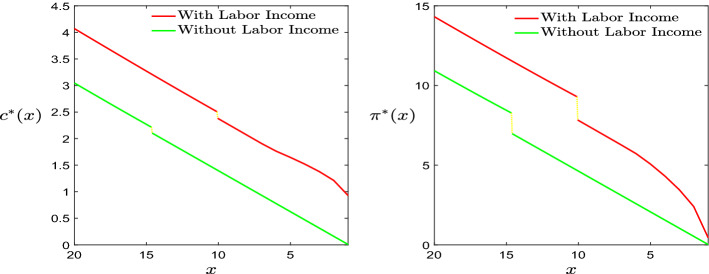
Influence of labour income

**Fig. 6:**
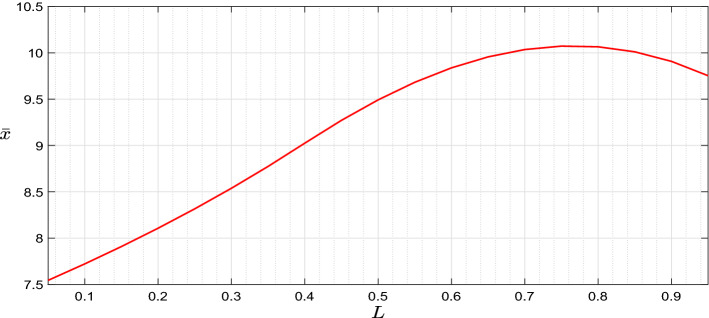
Optimal bankruptcy threshold w.r.t. maximum leisure rate (up) and wage rate (down)

*Influence of the labour income*: In order to investigate the influence of introducing the leisure rate as an additional control variable and thus the labour income, we first of all compare the results with the optimal bankruptcy model in Jeanblanc et al. (2004), therefore with full leisure and no labour income, to conduct the numerical analysis to discover the sensitivity with respect to the presence of leisure rate and labour income. Figure 5 shows that there exists a downward jumps of optimal control strategies for both full and selectable leisure models due to the shrinkage of wealth at the moment of declaring bankruptcy. Moreover, it can be observed that the optimal consumption and portfolio policies of the optimization problem introducing the leisure as a control variable always keep dominating the corresponding policies of the model with full leisure rate since the agent can earn the additional income from labour. Meanwhile, comparing the wealth levels corresponding to the downward jumps, we can observe that the agent with the full leisure rate tends to have a higher bankruptcy wealth threshold such that more wealth can be taken into the post-bankruptcy period to support the further consumption ($\bar{x}=10.0651$ for the “with labour income case”, $\bar{x}=14.6091$ for the “without labour income -full leisure- case”). Secondly, in Fig. 6 we discover the sensitivity of bankruptcy wealth threshold with respect to different values of *L* within the optimization model considering leisure selection.

Figure 6 shows that the bankruptcy wealth threshold is not a monotonic function of *L*; the relationship between these two variables works in a complex way and can be analysed briefly into two separated pieces: one piece is with a relatively low value of *L*, and another is with higher *L* value. Since *L* represents the upper boundary of the leisure variable *l*(*t*), $(\bar{L}-L)$ represents the minimum mandatory working rate. In the first part, the low enough value of *L* obliges the agent to allocate most of the time on work, thereby gaining enough labour income to afford the debt repayment. Hence, the agent prefers to set a smaller wealth threshold for bankruptcy to avoid encountering the wealth shrinkage. However, for the second part, the restriction on the optimal leisure choice caused by *L* becomes weaker as its value increases, the agent gains the utility from taking more leisure, which leads to a decreasing labour income, a decreasing consumption since leisure and consumption are substitute, and a corresponding lower bankruptcy wealth threshold.^2^

We also observe an inverse relationship between $\bar{x}$ and *w* ($\bar{x}=12.7564$ if $w=0.6$, $\bar{x}=12.1523$ if $w=0.8$, $\bar{x}=11.5521$ if $w=1$, and $\bar{x}=10.9552$ if $w=1.2$): with a lower wage rate *w*, the agent tends to set a higher critical wealth level $\bar{x}$ given the increase of the difficulties to repay the debt, and in order to retain more wealth for supporting the post-bankruptcy life.

## Conclusion

In this work the optimal consumption-portfolio-leisure and bankruptcy problem concerning a power utility function has been solved semi-analytically. By the Legendre–Fenchel transform, we have established the duality between the optimization problem with the individual’s shadow price problem, which results in a system of variational inequalities then enables us to obtain the closed-form solutions. The optimal wealth and control strategies are represented as functions of wealth’s dual process, *Z*(*t*). Then we have proved that the optimal policy for the agent is to file bankruptcy at the first hitting time of the optimal wealth process to a critical wealth level, which is the boundary separating the continuous and stopping regions of the corresponding stopping time model. We have also conducted the sensitivity analysis of this wealth threshold to critical parameters. The bankruptcy wealth threshold is an increasing function of both *d*, which can be treated as the benefit of declaring bankruptcy, and $\alpha$, with $1-\alpha$ representing the flexible cost of bankruptcy. Whereas, the non-monotonic relationship between the bankruptcy wealth threshold and *F* is because *F* performs a trade-off between the liquidity constraint boundary in the pre-bankruptcy period and the fixed cost of bankruptcy. Regarding the effect of labour income, we show that the bankruptcy wealth threshold is a concave function of the upper bound of the leisure rate, *L*, that is, it first increases and then decreases: a high value for *L* permits the agent to have large utility from leisure, while a low value of *L* “forces” the agent to get a large wage, even if low utility from leisure. Moreover, the bankruptcy wealth threshold is strictly decreasing for the wage rate since a worse economic situation requires more wealth to support the post-bankruptcy period.

## Supplementary Information

Below is the link to the electronic supplementary material.

Supplementary file 1 (pdf 440 KB)

## Footnotes

1 Administrative Office of the U.S. Courts, Table F-2- Bankruptcy Filings (December 31, 2003) [Online]. Available: https://www.uscourts.gov/statistics/table/f-2/bankruptcy-filings/2003/12/31.

2 Figure 4 shows that, given the same liquidity constraint, it is optimal to set a higher leisure rate in presence of a bankruptcy option with respect to the case where this option is not available. Figure 6 shows that if *L* is large, and therefore the minimum mandatory working rate $(\bar{L}-L)$ is small, the optimal bankruptcy barrier is in general higher with respect to the case with a large minimum mandatory working rate, and therefore it is optimal for the agent to file bankruptcy with a larger wealth. In a period where the Covid-19 pandemic has led to forced leisure situations, therefore increasing *L*, our model can help to understand the impact in the increase of the bankruptcy threshold, suggesting policy to prevent a massive use of the bankruptcy option by citizens, like redesigning the bankruptcy procedure decreasing $\alpha$.
