# Supplementary material for: Effect of labour income on the optimal bankruptcy problem
Source: Ann Oper Res. 2023 Jan 22:1–23. Online ahead of print. doi: 10.1007/s10479-023-05166-z (PMC9867836; doi:10.1007/s10479-023-05166-z)
Supplement: Supplementary file 1 — (pdf 440 KB) [file 10479_2023_5166_MOESM1_ESM.pdf]

Effect of Labour Income on the Optimal Bankruptcy Problem - Online Appendix

Guodong Ding[1], Daniele Marazzina[1,2]
[1] Department of Mathematics, Politecnico di Milano
I-20133, Milano, Italy
[2] Corresponding Author. E-mail: daniele.marazzina@polimi.it

A Appendix of Section 2

A.1 Proof of Lemma 2.1

In order to get rid of the constraint $0 \le l \le L$, we begin with the maximization of $\{u(c,l) - (c + wl)y\}$ with $l \in \mathbb{R}$. From the first-order derivative conditions with respect to c and l, we obtain the following equations

$$\begin{cases} l^{(1-\delta)(1-k)} c^{\delta(1-k)-1} = y, \\ \frac{1-\delta}{\delta} l^{(1-\delta)(1-k)-1} c^{\delta(1-k)} = wy. \end{cases} \tag{A.1}$$

The above system entails the optimal consumption and leisure policies as

$$\begin{cases} \hat{c} = y^{-\frac{1}{k}} \left(\frac{1-\delta}{\delta w}\right)^{\frac{(1-k)(1-\delta)}{k}}, \\ \hat{l} = y^{-\frac{1}{k}} \left(\frac{1-\delta}{\delta w}\right)^{\frac{1-\delta(1-k)}{k}}, \end{cases}$$

$y > 0$ guarantees the positive values of $\hat{c}$ and $\hat{l}$. Besides, with treating $\hat{c}$ and $\hat{l}$ as functions of y, we get

$$\tilde{u}'(y) = \frac{\partial u}{\partial \hat{c}} \frac{d\hat{c}}{dy} + \frac{\partial u}{\partial \hat{l}} \frac{d\hat{l}}{dy} - (\hat{c} + w\hat{l}) - \frac{d\hat{c}}{dy} y - wy \frac{d\hat{l}}{dy} = -(\hat{c} + w\hat{l}).$$

Then the remaining constraint of the convex dual transform of Equation (2.4) is $\hat{l} \le L$. Since

$$\hat{l} \le L \quad \Leftrightarrow \quad y \ge \left(\frac{1-\delta}{\delta w}\right)^{1-\delta(1-k)} L^{-k} \triangleq \tilde{y},$$

the optimal leisure plan $\hat{l}$ also satisfies the constraint $l \le L$ under the condition $y \ge \tilde{y}$. Conversely, this constraint comes into force to make the optimal leisure to be L for the interval $y < \tilde{y}$. Thereafter, we can summarize as follows,

$$\hat{l} = y^{-\frac{1}{k}} \left(\frac{1-\delta}{\delta w}\right)^{-\frac{\delta(1-k)-1}{k}} \mathbb{I}_{\{y \ge \tilde{y}\}} + L \mathbb{I}_{\{0 < y < \tilde{y}\}}. \tag{A.2}$$

The first equation in (A.1) implies the relationship between the optimal consumption and leisure, $\hat{c} = \left[y l^{-(1-k)(1-\delta)}\right]^{\frac{1}{\delta(1-k)-1}}$. Taking Equation (A.2) into this relationship, we obtain

$$\hat{c} = y^{-\frac{1}{k}} \left(\frac{1-\delta}{\delta w}\right)^{\frac{(1-k)(1-\delta)}{k}} \mathbb{I}_{\{y \ge \tilde{y}\}} + L^{-\frac{(1-k)(1-\delta)}{\delta(1-k)-1}} y^{\frac{1}{\delta(1-k)-1}} \mathbb{I}_{\{0 < y < \tilde{y}\}}.$$

Finally, we can deduce the convex dual transform $\tilde{u}(y)$ directly by substituting $\hat{c}$ and $\hat{l}$ into Equation (2.4).

A.2 Proof of Proposition 2.1

Referring to (Karatzas and Shreve, 1998b, Section 3.3, Remark 3.3), we first apply Itô's lemma to $\xi(t)X(t)$, $\forall t \in [0,\tau]$,

$$\begin{aligned} d\left(\xi(t)X(t)\right) &= \xi(t)dX(t) + X(t)d\xi(t) \\ &= -\xi(t)\left(c(t)+d+wl(t)-w\bar{L}\right)dt + \pi(t)\left(\mu-r\right)\xi(t)dt + \sigma\xi(t)\pi(t)dB(t) \\ &= -\xi(t)\left(c(t)+d+wl(t)-w\bar{L}\right)dt + +\sigma\xi(t)\pi(t)d\tilde{B}(t), \end{aligned}$$

in which $\tilde{B}(t)$ is the Brownian motion under the $\tilde{\mathbb{P}}$ measure mentioned in Equation (2.2). Taking the integral on both sides of the above equation from 0 to τ, we obtain

$$\int_0^\tau \xi(t)\left(c(t)+d+wl(t)-w\bar{L}\right)dt + \xi(\tau)X(\tau) = x + \int_0^\tau \sigma\xi(t)\pi(t)d\tilde{B}(t).$$

The left-hand side can be rewritten as

$$\int_0^\tau \xi(t)(c(t)+d+wl(t)-w\bar{L})dt+\xi(\tau)X(\tau)=\int_0^\tau \xi(t)(c(t)+wl(t))dt+\xi(\tau)\left(X(\tau)-\frac{d-w\bar{L}}{r}\right)+\frac{d-w\bar{L}}{r},$$

the condition $X(\tau) \geq F+\eta \geq \frac{d-w\bar{L}}{r}$ from the definition of admissible control set ensures that the left-hand side is bounded below by the constant $\frac{d-w\bar{L}}{r}$, so the Itô integral on the right-hand side is proved to be a $\tilde{\mathbb{P}}$-supermartingale by means of Fatou's lemma. Then, taking the expectation on both sides under the $\tilde{\mathbb{P}}$ measure, we have

$$\tilde{\mathbb{E}}\left[\int_0^\tau \xi(t)(c(t)+d+wl(t)-w\bar{L})dt + \xi(\tau)X(\tau)\right] = x + \tilde{\mathbb{E}}\left[\int_0^\tau \sigma\xi(t)\pi(t)d\tilde{B}(t)\right] \leq x,$$

which endows us with the desired budget constraint through converting the measure to $\mathbb{P}$,

$$\mathbb{E}\left[\int_0^\tau H(t)\left(c(t)+d+wl(t)-w\bar{L}\right)dt+H(\tau)X(\tau)\right]=\tilde{\mathbb{E}}\left[\int_0^\tau \xi(t)(c(t)+d+wl(t)-w\bar{L})dt+\xi(\tau)X(\tau)\right]\leq x.$$

B Appendix of Section 3

B.1 Proof of Theorem 3.1

Before proving Theorem 3.1, we insert a lemma which helps us to prove the theorem.

Lemma B.1. *For any given initial wealth $x \geq 0$, and any given and progressively measurable consumption and leisure processes, $c(t)$, $l(t)$, satisfying $\sup_{\tau\in\mathcal{T}} \mathbb{E}\left[\int_0^\tau H(t)(c(t)+wl(t)-w\bar{L})dt\right] \leq x$, with $\mathcal{T}$ standing for the set of $\mathbb{F}$-stopping times, there exists a portfolio process $\pi(t)$ making*

$$X^{x,c,\pi,l}(t) \geq 0, \quad \forall t \geq 0,$$

holds almost surely.

Proof. Referring to (He and Pagès, 1993, Appendix, Lemma 1), we first define a new process

$$K(t) \triangleq \int_0^t (c(s) + wl(s) - w\bar{L})H(s)ds, \quad \forall t \geq 0.$$

Proposition 3.1 implies $\mathbb{E}[K(t)] \leq x$, $\forall t \geq 0$, from which we can deduce that $K(t)$ is a uniformly integrable martingale, i.e., the collection $\{K(\tau)\}_{\tau \in \mathcal{T}}$ is uniformly integrable. Then, as in (Dellacherie and Meyer, 1982, Appendix I), there exists a Snell envelope of $K(t)$ denoted as $\bar{K}(t)$. It is a super-martingale under the $\mathbb{P}$ measure and satisfies

$$\bar{K}(0) = \sup_{\tau \in \mathcal{T}} \mathbb{E}[K(\tau)], \quad \bar{K}(\infty) = K(\infty).$$

By the Doob-Meyer Decomposition Theorem from (Karatzas and Shreve, 1998a, Section 1.4, Theorem 4.10), the super-martingale $\bar{K}(t)$ can be represented as

$$\bar{K}(t) = \bar{K}(0) + \bar{M}(t) - \bar{A}(t),$$

where $\bar{M}(t)$ is a uniformly integrable martingale under the $\mathbb{P}$ measure with the initial value $\bar{M}(0) = 0$, $\bar{A}(t)$ is an increasing process with the initial value $\bar{A}(0) = 0$. According to the Martingale Representation Theorem from (Björk, 2009, Section 11.1, Theorem 11.2), $\bar{M}(t)$ can be expressed as

$$\bar{M}(t) = \int_0^t \bar{\rho}(s)dB(s), \quad \forall t \geq 0,$$

with an $\mathbb{F}$-adapted process $\bar{\rho}(t)$ satisfying $\int_0^\infty \bar{\rho}^2(s)ds < \infty$ a.s.. Let us define a new process

$$\bar{X}(t) \triangleq \frac{1}{H(t)}\left[x - \bar{K}(0) + \bar{K}(t) - K(t) + \bar{A}(t)\right].$$

Based on the fact that $\bar{K}(0) = \sup_{\tau \in \mathcal{T}} \mathbb{E}[K(\tau)] = \sup_{\tau \in \mathcal{T}} \mathbb{E}\left[\int_0^\tau H(t)(c(t) + wl(t) - w\bar{L})dt\right] \leq x$, we can conclude that $\bar{X}(t)$ is a non-negative process with the initial wealth $\bar{X}(0) = x$. We express this process with the martingale $\bar{M}(t)$ as

$$\bar{X}(t) = \frac{1}{H(t)}\left[x + \int_0^t \bar{\rho}(s)dB(s) - K(t)\right] = \frac{1}{H(t)}\left[x + \int_0^t \bar{\rho}(s)dB(s) - \int_0^t (c(s) + wl(s) - w\bar{L})H(s)ds\right].$$

As for the dynamics of wealth process

$$dX^{x,c,\pi,l}(t) = rX^{x,c,\pi,l}(t)dt + \pi(t)(\mu - r)dt - c(t)dt + w(\bar{L} - l(t))dt + \sigma\pi(t)dB(t),$$

we implement Itô's lemma to $H(t)X^{x,c,\pi,l}(t)$ to get

$$d(H(t)X^{x,c,\pi,l}(t)) = -H(t)X^{x,c,\pi,l}(t)\theta dB(t) - (c(t) + wl(t) - w\bar{L})H(t)dt + \sigma\pi(t)H(t)dB(t).$$

If we take the portfolio strategy as $\pi(t) = \frac{\bar{\rho}(t)}{\sigma H(t)} + \frac{\theta X^{x,c,\pi,l}(t)}{\sigma}$, the wealth process is rewritten as $X^{x,c,\pi,l}(t) = \frac{1}{H(t)}\left[x + \int_0^t \bar{\rho}(s)dB(s) - \int_0^t (c(s) + wl(s) - w\bar{L})H(s)ds\right]$, which shows that $\bar{X}(t) = X^{x,c,\pi,l}(t)$, a.s.. The non-negativity of $\bar{X}(t)$ claims $X^{x,c,\pi,l}(t) \geq 0$, a.s., $\forall t \geq 0$. □

Then we move to the proof of the Duality Theorem 3.1. Following (He and Pagès, 1993, Section 4, Theorem 1), the proof mainly contains two aspects: the first part is to show the admissibility of $c^*(t)$ and $l^*(t)$, and the second part is to claim that they are the optimal consumption-leisure strategy to Problem (P_{PB}).
(1) We begin verifying that any consumption-leisure strategy satisfying

$$c^*(t) + wl^*(t) = -\tilde{u}'(\lambda^* e^{\gamma t} D^*_{PB}(t)H(t))$$

is admissible. Taking any stopping time τ from $\mathcal{T}$, which is the set of $\mathbb{F}$-stopping times, we can define a process

$$D^\epsilon(t) \triangleq D^*_{PB}(t) + \epsilon \mathbb{I}_{[0,\tau)}(t),$$

where ϵ a positive constant. It is evident that $D^\epsilon(t)$ is a non-negative, non-increasing, and progressively measurable process, that is, $D^\epsilon(t) \in \mathcal{D}$. Let us define a function

$$\mathfrak{L}(D(t)) \triangleq \mathbb{E}\left[\int_0^\infty e^{-\gamma t}(\tilde{u}(\lambda^* e^{\gamma t} D(t)H(t)) + w\bar{L}\lambda^* e^{\gamma t} D(t)H(t))dt\right] + \lambda^* x D(0).$$

Since $D^*_{PB}(t)$ is the optimal solution of Problem (S_{PB}), and $x \geq 0$, we get

$$\begin{aligned}\mathfrak{L}(D^*_{PB}(t)) &= \mathbb{E}\left[\int_0^\infty e^{-\gamma t}(\tilde{u}(\lambda^* e^{\gamma t} D^*_{PB}(t)H(t)) + w\bar{L}\lambda^* e^{\gamma t} D^*_{PB}(t)H(t))dt\right] + \lambda^* x D^*_{PB}(0)\\ &\leq \mathbb{E}\left[\int_0^\infty e^{-\gamma t}(\tilde{u}(\lambda^* e^{\gamma t} D^\epsilon(t)H(t)) + w\bar{L}\lambda^* e^{\gamma t} D^\epsilon(t)H(t))dt\right] + \lambda^* x (D^*_{PB}(0) + \epsilon)\\ &= \mathfrak{L}(D^\epsilon(t)), \quad \forall t \geq 0.\end{aligned}$$

The above inequalities give us $\limsup\limits_{\epsilon \downarrow 0} \frac{\mathfrak{L}(D^\epsilon(t)) - \mathfrak{L}(D^*_{PB}(t))}{\epsilon} \geq 0$ and

$$\limsup_{\epsilon \downarrow 0} \mathbb{E}\left[\int_0^\tau \left(e^{-\gamma t} \frac{\tilde{u}(\lambda^* e^{\gamma t} D^\epsilon(t)H(t)) - \tilde{u}(\lambda^* e^{\gamma t} D^*_{PB}(t)H(t))}{\epsilon} + w\bar{L}\lambda^* H(t)\right) dt\right] + \lambda^* x \geq 0.$$

The decreasing property of $\tilde{u}(\cdot)$ endows us with $\tilde{u}(\lambda^* e^{\gamma t} D^\epsilon(t)H(t)) \leq \tilde{u}(\lambda^* e^{\gamma t} D^*_{PB}(t)H(t))$. Applying Fatou's lemma, we have

$$\mathbb{E}\left[\int_0^\tau \lambda^* H(t)\tilde{u}'(\lambda^* e^{\gamma t} D^*_{PB}(t)H(t))dt\right] \geq \limsup_{\epsilon \downarrow 0} \mathbb{E}\left[\int_0^\tau e^{-\gamma t} \frac{\tilde{u}(\lambda^* e^{\gamma t} D^\epsilon(t)H(t)) - \tilde{u}(\lambda^* e^{\gamma t} D^*_{PB}(t)H(t))}{\epsilon} dt\right].$$

Because of $c^*(t) + wl^*(t) = -\tilde{u}'(\lambda^* e^{\gamma t} D^*_{PB}(t)H(t))$, we get $\mathbb{E}\left[\int_0^\tau H(t)(c^*(t) + wl^*(t) - w\bar{L})dt\right] \leq x$. Since τ can be any $\mathbb{F}$-stopping time in the set $\mathcal{T}$, there exists a portfolio strategy $\pi^*(t)$ that makes the corresponding wealth process satisfying $X^{x,c^*,\pi^*,l^*}(t) \geq 0$, $\forall t \geq 0$ based on the result from Lemma B.1.
(2) In this part, we claim that $c^*(t)$ and $l^*(t)$ are the optimal consumption and leisure to Problem (P_{PB}) under the liquidity constraint. Taking an arbitrary consumption strategy $\{c(t), \pi(t), l(t)\} \in \mathcal{A}_{PB}(x)$, the proof of Lemma B.1 guarantees that there exists a process $\zeta(t)$ satisfying

$$\int_0^t H(s)(c(s) + wl(s) - w\bar{L})ds + H(t)X^{x,c,\pi,l}(t) = x + \int_0^t \zeta(s)dB(s). \tag{B.1}$$

Since $X^{x,c,\pi,l}(t) \geq 0$ a.s., we obtain the following inequality with any process $D(t) \in \mathcal{D}$,

$$\int_0^T \int_0^t H(s)(c(s) + wl(s) - w\bar{L})dsdD(t) \geq \int_0^T \left[x + \int_0^t \zeta(s)dB(s)\right] dD(t),$$

where T is any time satisfying $T \geq t$. Because $D(t)$ is bounded variation, we can integrate by parts and get

$$\int_0^T D(s)H(s)(c(s) + wl(s) - w\bar{L})ds - \int_0^T D(s)\zeta(s)dB(s) \leq$$
$$D(0)x + D(T)\left[\int_0^T H(s)(c(s) + wl(s) - w\bar{L})ds - x - \int_0^T \zeta(s)dB(s)\right].$$

Taking the expectation under the $\mathbb{P}$ measure on both sides and replacing Equation (B.1), we obtain

$$\mathbb{E}\left[\int_0^T D(s)H(s)(c(s) + wl(s) - w\bar{L})ds\right] \leq D(0)x - \mathbb{E}\left[D(T)H(T)X^{x,c,\pi,l}(T)\right] \leq D(0)x,$$

then, by Lebesgue's Monotone Convergence Theorem, we have

$$\mathbb{E}\left[\int_0^\infty D(s)H(s)(c(s) + wl(s) - w\bar{L})ds\right] \leq D(0)x.$$

The above inequality keeps true for any admissible control strategy $c(t)$, $\pi(t)$, $l(t)$ and any non-negative, non-increasing process $D(t)$; furthermore, we will show that the inequality changes into equality with the given $c^*(t)$, $l^*(t)$ and $D^*_{PB}(t)$. We define a new process

$$\bar{D}^\epsilon(t) \triangleq D^*_{PB}(t)(1+\epsilon) \in \mathcal{D},$$

where ϵ is a small enough constant. Following the same argument as in the first part of the proof, we have $\mathfrak{L}(\bar{D}^\epsilon(t)) \geq \mathfrak{L}(D^*_{PB}(t))$ and

$$\limsup_{\epsilon\downarrow 0} \frac{\mathfrak{L}(\bar{D}^\epsilon(t)) - \mathfrak{L}(D^*_{PB}(t))}{\epsilon} = \limsup_{\epsilon\downarrow 0} \mathbb{E}\left[\int_0^\infty e^{-\gamma t} \frac{\tilde{u}(\lambda^* e^{\gamma t} D^*_{PB}(t)(1+\epsilon)H(t)) - \tilde{u}(\lambda^* e^{\gamma t} D^*_{PB}(t)H(t))}{\epsilon} dt\right.$$
$$\left. + \int_0^\infty w\bar{L}\lambda^* D^*_{PB}(t)H(t)dt\right] + \lambda^* x D^*_{PB}(0) \geq 0,$$

$$\liminf_{\epsilon\uparrow 0} \frac{\mathfrak{L}(\bar{D}^\epsilon(t)) - \mathfrak{L}(D^*_{PB}(t))}{\epsilon} = \liminf_{\epsilon\uparrow 0} \mathbb{E}\left[\int_0^\infty e^{-\gamma t} \frac{\tilde{u}(\lambda^* e^{\gamma t} D^*_{PB}(t)(1+\epsilon)H(t)) - \tilde{u}(\lambda^* e^{\gamma t} D^*_{PB}(t)H(t))}{\epsilon} dt\right.$$
$$\left. + \int_0^\infty w\bar{L}\lambda^* D^*_{PB}(t)H(t)dt\right] + \lambda^* x D^*_{PB}(0) \leq 0.$$

Applying Fatou's lemma, we obtain separately

$$\mathbb{E}\left[\int_0^\infty D^*_{PB}(t)H(t)(c^*(t) + wl^*(t) - w\bar{L})dt\right] \leq xD^*_{PB}(0),$$
$$\mathbb{E}\left[\int_0^\infty D^*_{PB}(t)H(t)(c^*(t) + wl^*(t) - w\bar{L})dt\right] \geq xD^*_{PB}(0),$$

which give us $\mathbb{E}\left[\int_0^\infty D^*_{PB}(t)H(t)(c^*(t)+wl^*(t)-w\bar{L})dt\right] = xD^*_{PB}(0)$. Subsequently, we define a new optimization problem named (P'_{PB}) as

$$\max_{c(t)\geq 0, l(t)\geq 0} \mathbb{E}\left[\int_0^\infty e^{-\gamma t}u(c(t),l(t))dt\right] \qquad (P'_{PB})$$

$$s.t. \quad \mathbb{E}\left[\int_0^\infty D^*_{PB}(t)H(t)(c(t)+wl(t)-w\bar{L})dt\right] \leq xD^*_{PB}(0).$$

We denote the optimal consumption solution of the above problem is $\tilde{c}^*(t)$. From the Lagrange method, we know that $\tilde{c}^*(t) + w\tilde{l}^*(t) = -\tilde{u}'(\tilde{\lambda}e^{\gamma t}D^*_{PB}(t)H(t))$, where $\tilde{\lambda} > 0$ is the Lagrange multiplier. The constraint of problem (P'_{PB}) takes equality when $\tilde{\lambda} = \lambda^*$. Then, the condition $c^*(t) + wl^*(t) = -\tilde{u}'(\lambda^* e^{\gamma t}D^*_{PB}(t)H(t))$ implies that $c^*(t)$ and $l^*(t)$ are the optimal control policies of Problem (P'_{PB}). Moreover, since the maximum utility of primal problem (P_{PB}) is upper bounded by the maximum utility of (P'_{PB}), we can conclude that $c^*(t)$ and $l^*(t)$ are also the optimal consumption and leisure solutions of Problem (P_{PB}).

B.2 Solutions of Variational Inequalities (3.4)

The solution is computed considering two cases: $0 < \tilde{y} \leq \hat{z}_{PB}$ and $0 < \hat{z}_{PB} < \tilde{y}$.

Case 1. $0 < \tilde{y} \leq \hat{z}_{PB}$**:** Condition $(V3)$ in (3.4) results in a differential equation,

$$-\gamma v_{PB}(z) + (\gamma - r)zv'_{PB}(z) + \frac{1}{2}\theta^2 z^2 v''_{PB}(z) + \tilde{u}(z) + w\bar{L}z = 0, \qquad 0 < z < \hat{z}_{PB}, \tag{B.2}$$

which has the solution

$$v_{PB}(z) = \begin{cases} B_{11,PB}z^{n_1} + B_{21,PB}z^{n_2} + \frac{A_1}{\Gamma_1}z^{\frac{\delta(1-k)}{\delta(1-k)-1}} + \frac{w(\bar{L}-L)}{r}z, & 0 < z < \tilde{y}, \\ B_{12,PB}z^{n_1} + B_{22,PB}z^{n_2} + \frac{A_2}{\Gamma_2}z^{-\frac{1-k}{k}} + \frac{w\bar{L}}{r}z, & \tilde{y} \leq z < \hat{z}_{PB}, \end{cases}$$

with the new defined constants

$$n_{1,2} \triangleq -\frac{\gamma - r - \frac{\theta^2}{2}}{\theta^2} \mp \sqrt{\left(\frac{\gamma - r - \frac{\theta^2}{2}}{\theta^2}\right)^2 + \frac{2\gamma}{\theta^2}}, \quad n_1 < 0, \quad n_2 > 1,$$

$$\Gamma_1 \triangleq \frac{\gamma - r\delta(1-k) - \frac{\theta^2}{2}\frac{\delta(1-k)}{1-\delta(1-k)}}{1-\delta(1-k)}, \quad \text{and} \quad \Gamma_2 \triangleq \frac{\gamma - r(1-k) - \frac{\theta^2}{2}\frac{1-k}{k}}{k}.$$

For avoiding the explosion of term z^{n_1} when z goes to 0, we set $B_{11,PB} = 0$. Then four parameters are left to be determined, which are $B_{21,PB}$, $B_{12,PB}$, $B_{22,PB}$ and $\hat{z}_{PB}$. To accomplish this task, we use the smooth conditions at $z = \tilde{y}$ and $z = \hat{z}_{PB}$ to construct a four-equation system:

- $\mathcal{C}^0$ condition at $z = \tilde{y}$

$$B_{21,PB}\tilde{y}^{n_2} + \frac{A_1}{\Gamma_1}\tilde{y}^{\frac{\delta(1-k)}{\delta(1-k)-1}} - \frac{wL}{r}\tilde{y} = B_{12,PB}\tilde{y}^{n_1} + B_{22,PB}\tilde{y}^{n_2} + \frac{A_2}{\Gamma_2}\tilde{y}^{-\frac{1-k}{k}}; \tag{B.3}$$

- $\mathcal{C}^1$ condition at $z=\tilde{y}$

$$n_2 B_{21,PB}\tilde{y}^{n_2-1}+\frac{\delta(1-k)}{\delta(1-k)-1}\frac{A_1}{\Gamma_1}\tilde{y}^{\frac{1}{\delta(1-k)-1}}-\frac{wL}{r}=n_1 B_{12,PB}\tilde{y}^{n_1-1}+n_2 B_{22,PB}\tilde{y}^{n_2-1}-\frac{1-k}{k}\frac{A_2}{\Gamma_2}\tilde{y}^{-\frac{1}{k}}; \tag{B.4}$$

- $\mathcal{C}^1$ condition at $z=\hat{z}_{PB}$

$$n_1 B_{12,PB}\hat{z}_{PB}^{n_1-1}+n_2 B_{22,PB}\hat{z}_{PB}^{n_2-1}-\frac{1-k}{k}\frac{A_2}{\Gamma_2}\hat{z}_{PB}^{-\frac{1}{k}}+\frac{w\bar{L}}{r}=0; \tag{B.5}$$

- $\mathcal{C}^2$ condition at $z=\hat{z}_{PB}$

$$n_1(n_1-1)B_{12,PB}\hat{z}_{PB}^{n_1-2}+n_2(n_2-1)B_{22,PB}\hat{z}_{PB}^{n_2-2}+\frac{1-k}{k^2}\frac{A_2}{\Gamma_2}\hat{z}_{PB}^{-\frac{1+k}{k}}=0. \tag{B.6}$$

Multiplying (B.4) by $\tilde{y}$ then subtracting it from (B.3), we can get the expression of $B_{21,PB}\tilde{y}^{n_2}$ as

$$B_{21,PB}\tilde{y}^{n_2}=-\frac{1}{(\delta(1-k)-1)(n_2-1)}\frac{A_1}{\Gamma_1}\tilde{y}^{\frac{\delta(1-k)}{\delta(1-k)-1}}+\frac{n_1-1}{n_2-1}B_{12,PB}\tilde{y}^{n_1}+B_{22,PB}\tilde{y}^{n_2}-\frac{1}{k(n_2-1)}\frac{A_2}{\Gamma_2}\tilde{y}^{-\frac{1-k}{k}}. \tag{B.7}$$

Replacing the above equation back to (B.3), the exact value of $B_{12,PB}$ is uniquely solved, that is,

$$B_{12,PB}=-\frac{\delta(1-k)n_2-\delta(1-k)-n_2}{(\delta(1-k)-1)(n_1-n_2)}\frac{A_1}{\Gamma_1}\tilde{y}^{\frac{\delta(1-k)}{\delta(1-k)-1}-n_1}+\frac{1+k(n_2-1)}{k(n_1-n_2)}\frac{A_2}{\Gamma_2}\tilde{y}^{-\frac{1-k}{k}-n_1}+\frac{n_2-1}{n_1-n_2}\frac{wL}{r}\tilde{y}^{1-n_1}.$$

Multiplying (B.6) by $\hat{z}_{PB}$ then adding with (B.5), we get the expression of $B_{12,PB}\hat{z}_{PB}^{n_1-1}$ as

$$B_{12,PB}\hat{z}_{PB}^{n_1-1}=-\left(\frac{n_2}{n_1}\right)^2 B_{22,PB}\hat{z}_{PB}^{n_2-1}-\left(\frac{1-k}{n_1 k}\right)^2\frac{A_2}{\Gamma_2}\hat{z}_{PB}^{-\frac{1}{k}}-\frac{1}{n_1^2}\frac{w\bar{L}}{r}.$$

Replacing it into (B.5), we obtain

$$B_{22,PB}\hat{z}_{PB}^{n_2-1}=\frac{1-k}{k^2}\frac{1-k+n_1k}{n_2(n_1-n_2)}\frac{A_2}{\Gamma_2}\hat{z}_{PB}^{-\frac{1}{k}}-\frac{n_1-1}{n_2(n_1-n_2)}\frac{w\bar{L}}{r}. \tag{B.8}$$

Thereafter, $B_{12,PB}$ is obtained by substituting the above expression:

$$B_{12,PB}=-\frac{1-k}{k^2}\frac{1-k+n_2k}{n_1(n_1-n_2)}\frac{A_2}{\Gamma_2}\hat{z}_{PB}^{-\frac{1-k}{k}-n_1}+\frac{n_2-1}{n_1(n_1-n_2)}\frac{w\bar{L}}{r}\hat{z}_{PB}^{1-n_1}.$$

Since the value of $B_{12,PB}$ is known, the above entails us to get the value of $\hat{z}_{PB}$. To show the uniqueness and existence of $\hat{z}_{PB}$, we first define the function

$$f(z)\triangleq-\frac{1-k}{k^2}\frac{1-k+n_2k}{n_1(n_1-n_2)}\frac{A_2}{\Gamma_2}z^{-\frac{1-k}{k}-n_1}+\frac{n_2-1}{n_1(n_1-n_2)}\frac{w\bar{L}}{r}z^{1-n_1}-B_{12,PB},\quad \forall z>0.$$

Then the solution $\hat{z}_{PB}$ is transformed into the zero of function $f(z)$. Taking the first derivative of $f(z)$, we get

$$f'(z)=\frac{1-k}{k^3}\frac{(1-k+n_1k)(1-k+n_2k)}{n_1(n_1-n_2)}\frac{A_2}{\Gamma_2}z^{-\frac{1}{k}-n_1}+\frac{(n_2-1)(1-n_1)}{n_1(n_1-n_2)}\frac{w\bar{L}}{r}z^{-n_1}.$$

Considering the conditions, $n_1+n_2=-\frac{\gamma-r-\frac{\theta^2}{2}}{\frac{\theta^2}{2}}$, $n_1n_2=-\frac{\gamma}{\frac{\theta^2}{2}}$ and $r=\frac{\theta^2}{2}(n_2-1)(1-n_1)$, the inequality $f'(z)>0$ can be solved as follows:

$$\begin{aligned} z &> \left[\frac{k^3}{k-1}\frac{(n_2-1)(1-n_1)}{(1-k+n_1k)(1-k+n_2k)}\frac{\Gamma_2}{A_2}\frac{w\bar{L}}{r}\right]^{-k} \\ &= \left[\frac{k(\delta-1)}{\frac{\theta^2}{2}}\frac{\gamma-r(1-k)-\frac{\theta^2}{2}\frac{1-k}{k}}{(1-k+n_1k)(1-k+n_2k)}\left(\frac{1-\delta}{\delta w}\right)^{\frac{\delta(1-k)-1}{k}}\bar{L}\right]^{-k} \\ &= \left[(\delta-1)\frac{\gamma-r(1-k)-\frac{\theta^2}{2}\frac{1-k}{k}}{-\gamma+r(1-k)+\frac{\theta^2}{2}\frac{1-k}{k}}\left(\frac{1-\delta}{\delta w}\right)^{\frac{\delta(1-k)-1}{k}}\bar{L}\right]^{-k} \\ &= (1-\delta)^{-k}\left(\frac{1-\delta}{\delta w}\right)^{1-\delta(1-k)}\bar{L}^{-k}, \end{aligned}$$

which indicates that $f(z)$ is decreasing on $\left(0,(1-\delta)^{-k}\left(\frac{1-\delta}{\delta w}\right)^{1-\delta(1-k)}\bar{L}^{-k}\right)$, and then increasing on $\left((1-\delta)^{-k}\left(\frac{1-\delta}{\delta w}\right)^{1-\delta(1-k)}\bar{L}^{-k},\infty\right)$. Then we determine the sign of $f(\tilde{y})$ with basic calculations:

$$\begin{aligned} f(\tilde{y})\tilde{y}^{n_1} =& -\frac{1-k}{k^2}\frac{1-k+n_2k}{n_1(n_1-n_2)}\frac{A_2}{\Gamma_2}\tilde{y}^{-\frac{1-k}{k}}+\frac{n_2-1}{n_1(n_1-n_2)}\frac{w\bar{L}}{r}\tilde{y}+\frac{\delta(1-k)n_2-\delta(1-k)-n_2}{(\delta(1-k)-1)(n_1-n_2)}\frac{A_1}{\Gamma_1}\tilde{y}^{\frac{\delta(1-k)}{\delta(1-k)-1}} \\ &-\frac{1-k+n_2k}{k(n_1-n_2)}\frac{A_2}{\Gamma_2}\tilde{y}^{-\frac{1-k}{k}}-\frac{n_2-1}{n_1-n_2}\frac{wL}{r}\tilde{y} \\ =& \frac{1}{\frac{\theta^2}{2}n_1(n_1-n_2)}A_2\tilde{y}^{-\frac{1-k}{k}}+\frac{n_2-1}{n_1(n_1-n_2)}\frac{w\bar{L}}{r}\tilde{y}+\frac{\delta(1-k)n_2-\delta(1-k)-n_2}{(\delta(1-k)-1)(n_1-n_2)}\frac{A_1}{\Gamma_1}\tilde{y}^{\frac{\delta(1-k)}{\delta(1-k)-1}} \\ &-\frac{n_2-1}{n_1-n_2}\frac{wL}{r}\tilde{y} \\ =& \frac{1}{\frac{\theta^2}{2}n_1(n_1-n_2)}A_2\tilde{y}^{-\frac{1-k}{k}}+\frac{n_2-1}{n_1(n_1-n_2)}\frac{w\bar{L}}{r}\tilde{y}-\frac{\delta(1-k)}{(1-\delta(1-k))^2}\frac{\delta(1-k)+n_2(1-\delta(1-k))}{n_1(n_1-n_2)}\frac{A_1}{\Gamma_1}\tilde{y}^{\frac{\delta(1-k)}{\delta(1-k)-1}} \\ &-\frac{1}{\frac{\theta^2}{2}n_1(n_1-n_2)}A_1\tilde{y}^{\frac{\delta(1-k)}{\delta(1-k)-1}}-\frac{n_2-1}{n_1-n_2}\frac{wL}{r}\tilde{y}. \end{aligned}$$

Since $A_1\tilde{y}^{\frac{\delta(1-k)}{\delta(1-k)-1}}-wL\tilde{y}=A_2\tilde{y}^{-\frac{1-k}{k}}$ and given the inequality

$$\begin{aligned} \frac{(1-n_2)\left(\gamma-r\delta(1-k)-\frac{\theta^2}{2}\frac{\delta(1-k)}{1-\delta(1-k)}\right)}{n_2(\delta(1-k)-1)-\delta(1-k)} &= \frac{r\left(\gamma-r\delta(1-k)-\frac{\theta^2}{2}\frac{\delta(1-k)}{1-\delta(1-k)}\right)}{\frac{\theta^2}{2}n_2(n_1-1)(\delta(1-k)-1)-\frac{\theta^2}{2}(n_1-1)\delta(1-k)} \\ &= r\frac{\gamma-r\delta(1-k)-\frac{\theta^2}{2}\frac{\delta(1-k)}{1-\delta(1-k)}}{\gamma-r\delta(1-k)+\frac{\theta^2}{2}n_2}<r, \end{aligned} \tag{B.9}$$

$f(\tilde{y})\tilde{y}^{n_1}$ can be rewritten as,

$$
\begin{aligned}
f(\tilde{y})\tilde{y}^{n_1} &= -\frac{\delta(1-k)}{(1-\delta(1-k))^2}\frac{\delta(1-k)+n_2(1-\delta(1-k))}{n_1(n_1-n_2)}\frac{A_1}{\Gamma_1}\tilde{y}^{\frac{\delta(1-k)}{\delta(1-k)-1}}+\frac{n_2-1}{n_1(n_1-n_2)}\frac{w\bar{L}}{r}\tilde{y}-\frac{n_2-1}{n_1(n_1-n_2)}\frac{wL}{r}\tilde{y} \\
&< \frac{1-n_2}{n_1(n_1-n_2)}\frac{\delta}{1-\delta}\frac{wL}{r}\tilde{y} + \frac{n_2-1}{n_1(n_1-n_2)}\frac{w\bar{L}}{r}\tilde{y} - \frac{n_2-1}{n_1(n_1-n_2)}\frac{wL}{r}\tilde{y} \\
&= \frac{n_2-1}{n_1(n_1-n_2)}\frac{w\tilde{y}}{r}\left(\bar{L} - \frac{L}{1-\delta}\right).
\end{aligned}
$$

Therefore, $\bar{L}(1-\delta) \leq L$ is a sufficient condition for $f(\tilde{y}) < 0$. Considering $\lim_{z\to\infty} f(z) = \infty$, we can conclude that there exists a unique solution of $\hat{z}_{PB}$, satisfying $0 < \tilde{y} \leq \hat{z}_{PB}$, under the condition $\bar{L}(1-\delta) \leq L$. Moreover, in the next section, we also prove that the opposite condition $\bar{L}(1-\delta) > L$ is necessary for Case 2. $0 < \hat{z}_{PB} < \tilde{y}$. Then $B_{22,PB}$ is also explicitly solved by taking $\hat{z}_{PB}$ into (B.8). Finally, taking the solutions of $B_{12,PB}$, $B_{22,PB}$ into (B.7), the last remaining parameter, $B_{21,PB}$, is obtained. We will remove the computation details for the other cases of variational inequalities, since the same technique is applied.

Case 2. $0 < \hat{z}_{PB} < \tilde{y}$**:** As in the previous case, we first handle Condition $(V3)$ in (3.4). Recalling Lemma 2.1, the corresponding interval $0 < z < \hat{z}_{PB}$ restricts the function $\tilde{u}(z)$ only takes the form $\tilde{u}(z) = A_1 z^{\frac{\delta(1-k)}{\delta(1-k)-1}} - wLz$, which is identical with the piece of $0 < z < \tilde{y}$ in Case 1. Hence, the differential equation from $(V3)$ has the same solution, only changing the parameters' notations from $B_{11,PB}$ to $B_{1,PB}$, and $B_{21,PB}$ to $B_{2,PB}$, that is,

$$
v_{PB}(z) = B_{1,PB}z^{n_1} + B_{2,PB}z^{n_2} + \frac{A_1}{\Gamma_1}z^{\frac{\delta(1-k)}{\delta(1-k)-1}} + \frac{w(\bar{L}-L)}{r}z, \quad 0 < z < \hat{z}_{PB}.
$$

We set the coefficient $B_{1,PB} = 0$ for the reason that the term z^{n_1} goes to ∞ as z approaches 0, which violates the boundedness assumption of $v_{PB}(z)$. Subsequently, using the smooth condition at $z = \hat{z}_{PB}$, a two-equation system is established to determine the exact values of $B_{2,PB}$ and $\hat{z}_{PB}$:

- $\mathcal{C}^1$ condition at $z = \hat{z}_{PB}$

$$
n_2 B_{2,PB}\hat{z}_{PB}^{n_2-1} + \frac{\delta(1-k)}{\delta(1-k)-1}\frac{A_1}{\Gamma_1}\hat{z}_{PB}^{\frac{1}{\delta(1-k)-1}} + \frac{w(\bar{L}-L)}{r} = 0; \tag{B.10}
$$

- $\mathcal{C}^2$ condition at $z = \hat{z}_{PB}$

$$
n_2(n_2-1)B_{2,PB}\hat{z}_{PB}^{n_2-2} + \frac{\delta(1-k)}{(\delta(1-k)-1)^2}\frac{A_1}{\Gamma_1}\hat{z}_{PB}^{\frac{2-\delta(1-k)}{\delta(1-k)-1}} = 0. \tag{B.11}
$$

Multiplying Equation (B.11) with $\hat{z}_{PB}$ then adding with (B.10), we have

$$
B_{2,PB}\hat{z}_{PB}^{n_2-1} = -\left(\frac{\delta(1-k)}{n_2(\delta(1-k)-1)}\right)^2\frac{A_1}{\Gamma_1}\hat{z}_{PB}^{\frac{1}{\delta(1-k)-1}} - \frac{1}{n_2^2}\frac{w(\bar{L}-L)}{r}. \tag{B.12}
$$

Substituting $B_{2,PB}\hat{z}_{PB}^{n_2-1}$ into Equation (B.10), $\hat{z}_{PB}$ is uniquely determined

$$
\hat{z}_{PB} = \left[\frac{(n_2-1)(\delta(1-k)-1)}{n_2\delta(1-k)-n_2-\delta(1-k)}L^{\frac{(1-k)(1-\delta)}{\delta(1-k)-1}}K_1^{-1}\frac{w(\bar{L}-L)}{r}\right]^{\delta(1-k)-1},
$$

and $\bar{L}(1-\delta) > L$ is proved to be necessary for $\hat{z}_{PB} < \tilde{y}$ using the inequality (B.9). Then, B_2 is obtained by substituting the expression of $\hat{z}_{PB}$ back to Equation (B.12)

$$B_{2,PB} = -\left(\frac{\delta(1-k)}{n_2(\delta(1-k)-1)}\right)^2 \frac{A_1}{\Gamma_1}\hat{z}_{PB}^{\frac{\delta(1-k)}{\delta(1-k)-1}-n_2} - \frac{1}{n_2^2}\frac{w(\bar{L}-L)}{r}\hat{z}_{PB}^{1-n_2}.$$

C Appendix of Section 4

C.1 Proof of Proposition 4.1

Let us first introduce a process $M_\tau(t) \triangleq H(t)X(t) + \int_0^t \left(c(s)+d+wl(s)-w\bar{L}\right) H(s)ds$, $\forall t \in [0,\tau]$. Following (Karatzas and Shreve, 1998b, Section 3.9, Theorem 9.4), we firstly claim that $M_\tau(t)$ is a $\mathbb{P}$-martingale. Let $c(t)$ and $l(t)$ be the consumption and leisure processes such that

$$\mathbb{E}\left[\int_0^\infty H(s)\left(c(s)+d+wl(s)-w\bar{L}\right)ds\right] = x.$$

For any fixed stopping time $\tau \in \mathcal{T}$, we define $\zeta(\tau) \triangleq \frac{1}{H(\tau)}\mathbb{E}\left[\int_\tau^\infty H(s)\left(c(s)+d+wl(s)-w\bar{L}\right)ds\middle|\mathcal{F}_\tau\right]$. Then we have $x = \mathbb{E}\left[\int_0^\tau H(s)\left(c(s)+d+wl(s)-w\bar{L}\right)ds + H(\tau)\zeta(\tau)\right]$. (Karatzas and Shreve, 1998b, Section 3.3, Theorem 3.5) implies that there exists a portfolio process $\pi_\tau = \{\pi_\tau(t) : 0 \le t \le \tau\}$ satisfying $\zeta(\tau) = X^{x,c,\pi_\tau,l}(\tau)$. Moreover, since

$$\begin{aligned}
\mathbb{E}\left[M_\tau(\tau)\right] &= \mathbb{E}\left[H(\tau)X^{x,c,\pi_\tau,l}(\tau)\right] + \mathbb{E}\left[\int_0^\tau H(s)\left(c(s)+d+wl(s)-w\bar{L}\right)ds\right] \\
&= \mathbb{E}\left[H(\tau)\zeta(\tau)\right] + \mathbb{E}\left[\int_0^\tau H(s)\left(c(s)+d+wl(s)-w\bar{L}\right)ds\right] \\
&= \mathbb{E}\left[\mathbb{E}\left[\int_\tau^\infty H(s)\left(c(s)+d+wl(s)-w\bar{L}\right)ds\middle|\mathcal{F}_\tau\right] + \int_0^\tau H(s)\left(c(s)+d+wl(s)-w\bar{L}\right)ds\right] \\
&= x = M_\tau(0),
\end{aligned}$$

the process $\{M_\tau(t) : 0 \le t \le \tau\}$ is a $\mathbb{P}$-martingale. Combined with the constraint $X(t) \ge F + \eta$, $\forall t \in [0,\tau]$, we have

$$\mathbb{E}\left[H(\tau)X(\tau) + \int_0^\tau H(s)\left(c(s)+d+wl(s)-w\bar{L}\right)ds\middle|\mathcal{F}_t\right] = H(t)X(t) + \int_0^t H(s)\left(c(s)+d+wl(s)-w\bar{L}\right)ds,$$

then,

$$\mathbb{E}\left[\int_t^\tau \frac{H(s)}{H(t)}\left(c(s)+d+wl(s)-w\bar{L}\right)ds + \frac{H(\tau)}{H(t)}X(\tau)\middle|\mathcal{F}_t\right] = X(t) \ge F+\eta, \quad \forall t \in [0,\tau].$$

C.2 Proof of Theorem 4.1

The proof here is consistent with Appendix B.1, but some modification is needed due to the stop-time embedding. We first introduce a lemma, which will be used in the proof of the duality theorem.

Lemma C.1. *For any given initial wealth $x \geq F+\eta$, any $\mathbb{F}$-stopping time τ with $\mathbb{P}(\tau<\infty)=1$, any $\mathcal{F}_\tau$-measurable random variable Q with $\mathbb{P}(Q \geq F+\eta)=1$, and any given progressively measurable consumption and leisure processes $c(t) \geq 0$, $0 \leq l(t) \leq L$, satisfying*

$$\sup_{\tilde{\tau}\in\mathcal{S}}\mathbb{E}\left[-\int_{\tilde{\tau}}^{\tau} H(t)(c(t)+wl(t)-w\bar{L}+d)dt-H(\tau)Q\right] \leq -(F+\eta),$$

where $\mathcal{S}$ stands for the set of $\mathbb{F}$-stopping times before the fixed stopping time τ, and

$$\mathbb{E}\left[\int_0^{\tau} H(t)\left(c(t)+wl(t)-w\bar{L}+d\right)dt+H(\tau)Q\right]=x,$$

there exists a portfolio process $\pi(t)$ making $X^{x,c,\pi,l}(t) \geq F+\eta$, $\forall t \in [0,\tau]$, and $X^{x,c,\pi,l}(\tau)=Q$ hold almost surely.

Proof. Following similar argument with (He and Pagès, 1993, Appendix, Lemma 1), we first define a new process

$$K(t) \triangleq -\int_t^{\tau} H(s)(c(s)+wl(s)-w\bar{L}+d)ds - H(\tau)Q, \quad \forall t \in [0,\tau].$$

From the properties of processes $c(t)$, $l(t)$ and $H(t)$, it can be observed that $\mathbb{E}[K(t)] < \infty$, which implies $\{K(\tilde{\tau})\}_{\tilde{\tau}\in\mathcal{S}}$ is uniformly integrable. Therefore, there exists a Snell envelope of $K(t)$ denoted as $\bar{K}(t)$. It is a super-martingale under the $\mathbb{P}$ measure and satisfies

$$\bar{K}(0) = \sup_{\tilde{\tau}\in\mathcal{S}} \mathbb{E}[K(\tilde{\tau})], \quad \text{and} \quad \bar{K}(\tau) = K(\tau).$$

By the Doob-Meyer Decomposition Theorem from (Karatzas and Shreve, 1998a, Section 1.4, Theorem 4.10), the super-martingale $\bar{K}(t)$ can be decomposed into

$$\bar{K}(t) = \bar{K}(0) + \bar{M}(t) - \bar{A}(t),$$

where $\bar{M}(t)$ is a uniformly integrable martingale under the $\mathbb{P}$ measure with the initial value $\bar{M}(0) = 0$, $\bar{A}(t)$ is a strictly increasing process with the initial value $\bar{A}(0) = 0$. According to the Martingale Representation Theorem from (Björk, 2009, Section 11.1, Theorem 11.2), $\bar{M}(t)$ can be expressed as

$$\bar{M}(t) = \int_0^t \bar{\rho}(s)dB(s), \quad \forall t \in [0,\tau],$$

with an $\mathbb{F}$-adapted process $\bar{\rho}(t)$ satisfying $\int_0^\infty \bar{\rho}^2(s)ds < \infty$ a.s.. Let us define a new process

$$\bar{X}(t) \triangleq \frac{1}{H(t)}\mathbb{E}\left[-\bar{K}(0) + \bar{K}(t) - K(t) + \bar{A}(t) - \bar{M}(\tau)|\mathcal{F}_t\right] - F - \eta.$$

It can be verified that

$$\bar{X}(\tau) = Q - F - \eta, \quad \text{and} \quad \bar{X}(0) = x - F - \eta,$$

using the condition $\mathbb{E}\left[\int_0^\tau H(t)\left(c(t)+wl(t)-w\bar{L}+d\right)dt + H(\tau)Q\right] = x$, and the martingale property of $\bar{M}(t)$. Further, because of $\bar{K}(0) = \sup_{\tilde{\tau}\in\mathcal{S}} \mathbb{E}[K(\tilde{\tau})]$, we can prove that $\bar{K}(0) \geq K(t)$, $\forall t \in [0,\tau]$

by constructing a contradiction. Let us assume that $\tilde{\tau}^*$ attains the supremum within the expression of $\bar{K}(0)$, i.e., $\bar{K}(0) = K(\tilde{\tau}^*)$, and introduce a stopping time as $\bar{\tau} \triangleq \inf\{0 \le t \le \tau : K(t) > K(\tilde{\tau}^*)\}$. Since $\bar{\tau} \in \mathcal{S}$, we have $K(\bar{\tau}) \le K(\tilde{\tau}^*)$, which is contradictory to the definition of $\bar{\tau}$. Then, based on the fact that $\bar{X}(t) = \frac{1}{H(t)}\mathbb{E}[-\bar{K}(0)|\mathcal{F}_t] + \frac{1}{H(t)}\mathbb{E}[\bar{K}(0) - K(t)|\mathcal{F}_t] - F - \eta$, we can conclude that $\bar{X}(t) \ge 0$, a.s.. Additionally, the process $\bar{X}(t)$ is re-expressed in terms of the martingale $\bar{M}(t)$ as

$$\bar{X}(t)=\frac{1}{H(t)}\mathbb{E}\left[\int_t^\tau H(s)(c(s)+wl(s)-w\bar{L}+d)ds+H(\tau)Q-\int_t^\tau \bar{\rho}(s)dB(s)\middle|\mathcal{F}_t\right]-F-\eta. \tag{C.1}$$

As for the wealth process

$$\begin{cases} dX^{x,c,\pi,l}(t) = rX^{x,c,\pi,l}dt + \pi(t)(\mu - r)dt - (c(t) + wl(t) - w\bar{L} + d)dt + \sigma\pi(t)dB(t), \\ X^{x,c,\pi,l}(0) = x, \end{cases}$$

by implementing Itô's lemma to $H(t)X^{x,c,\pi,l}(t)$ and adopting the portfolio strategy as

$$\pi(t) = \frac{\bar{\rho}(t)}{\sigma H(t)} + \frac{\theta X^{x,c,\pi,l}(t)}{\sigma},$$

it is rewritten as $d(H(t)X^{x,c.\pi,l}(t)) = \bar{\rho}(t)dB(t) - (c(t) + wl(t) - w\bar{L} + d)H(t)dt$. Taking the integral from t to τ, and then the conditional expectation w.r.t. $\mathcal{F}_t$ on both sides of the above equation, we obtain

$$X^{x,c,\pi,l}(t)=\frac{1}{H(t)}\mathbb{E}\left[\int_t^\tau H(s)(c(s)+wl(s)-w\bar{L}+d)ds+H(\tau)X^{x,c,\pi,l}(\tau)-\int_t^\tau \bar{\rho}(s)dB(s)|\mathcal{F}_t\right].$$

Since $X^{x,c,\pi,l}(0)=x=\mathbb{E}\left[\int_0^\tau H(t)(c(t)+wl(t)-w\bar{L}+d)dt+H(\tau)X^{x,c,\pi,l}(\tau)\right]$, we have $X^{x,c,\pi,l}(\tau)=Q$, a.s.. Finally, through comparing the process of $X^{x,c,\pi,l}(t)$ with Equation (C.1),

$$\bar{X}(t)=X^{x,c,\pi}(t)-F-\eta, \quad a.s.$$

is observed. The non-negativity of $\bar{X}(t)$ claims $X^{x,c,\pi,l}(t)\ge F+\eta$, a.s., $\forall t \in [0,\tau]$. □

With the aid of the above lemma, we can complete the statement and proof of Duality Theorem 4.1. Referring to (He and Pagès, 1993, Section 4, Theorem 1), the proof procedure is divided into two aspects: the first part is focused on the admissibility of $c^*(t)$ and $l^*(t)$, and the second part is revolved around claiming that $c^*(t)$ and $l^*(t)$ are the optimal consumption-leisure strategy to the primal optimization problem.

(1) We begin verifying that any consumption-leisure strategy satisfying

$$c^*(t) + wl^*(t) = -\tilde{u}'(\lambda^* e^{\gamma t} D^*(t)H(t)), \quad \text{and} \quad X^{x,c^*,\pi^*,l^*}(\tau) = -\tilde{U}'(\lambda^* e^{\gamma\tau} D^*(\tau)H(\tau)),$$

is admissible. Taking any stopping time $\tilde{\tau}$ from $\mathcal{S}$, we can define a process $D^\epsilon(t) \triangleq D^*(t)+\epsilon\mathbb{I}_{[0,\tilde{\tau})}(t)$, where ϵ is a positive constant. It is evident that $D^\epsilon(t)$ is a non-negative, non-increasing and progressively measurable process, that is, $D^\epsilon(t) \in \mathcal{D}$. Let us define a function

$$\begin{aligned}\mathfrak{L}(D(t)) \triangleq \mathbb{E}&\left[\int_0^\tau e^{-\gamma t}\left(\tilde{u}(\lambda^* D(t)e^{\gamma t}H(t))-(d-w\bar{L})\lambda^* e^{\gamma t}D(t)H(t)\right)dt+e^{-\gamma\tau}\tilde{U}(\lambda^* D(\tau)e^{\gamma\tau}H(\tau))\right]\\ &+\lambda^*\mathbb{E}\left[\int_0^\tau (F+\eta)H(t)dD(t)\right]+\lambda^*(x-(F+\eta))D(0).\end{aligned}$$

Considering $D^*(t)$ is the optimal solution of problem (S_τ) and the fact $x \geq F+\eta$, we obtain

$$\begin{aligned}
\mathfrak{L}(D^*(t)) =& \mathbb{E}\left[\int_0^\tau e^{-\gamma t}\left(\tilde{u}(\lambda^* D^*(t)e^{\gamma t}H(t)) - (d-w\bar{L})\lambda^* e^{\gamma t}D^*(t)H(t)\right)dt + e^{-\gamma\tau}\tilde{U}(\lambda^* D^*(\tau)e^{\gamma\tau}H(\tau))\right]\\
&+ \lambda^*\mathbb{E}\left[\int_0^\tau (F+\eta)H(t)dD^*(t)\right] + \lambda^*(x-(F+\eta))D^*(0)\\
\leq& \mathbb{E}\left[\int_0^\tau e^{-\gamma t}\left(\tilde{u}(\lambda^* D^\epsilon(t)e^{\gamma t}H(t)) - (d-w\bar{L})\lambda^* e^{\gamma t}D^\epsilon(t)H(t)\right)dt + e^{-\gamma\tau}\tilde{U}(\lambda^* D^\epsilon(\tau)e^{\gamma\tau}H(\tau))\right]\\
&+ \lambda^*\mathbb{E}\left[\int_0^\tau (F+\eta)H(t)dD^\epsilon(t)\right] + \lambda^*(x-(F+\eta))(D^*(0)+\epsilon)\\
=& \mathfrak{L}(D^\epsilon(t)), \quad \forall t \geq\in [0,\tau].
\end{aligned}$$

The above inequalities give us $\limsup_{\epsilon\downarrow 0} \frac{\mathfrak{L}(D^\epsilon(t))-\mathfrak{L}(D^*(t))}{\epsilon} \geq 0$, combining with $dD^\epsilon(t) = dD^*(t)$, $D^\epsilon(\tau) = D^*(\tau)$, we have

$$\limsup_{\epsilon\downarrow 0}\mathbb{E}\left[\int_0^{\tilde{\tau}}\left(e^{-\gamma t}\frac{\tilde{u}(\lambda^* e^{\gamma t}D^\epsilon(t)H(t)) - \tilde{u}(\lambda^* e^{\gamma t}D^*(t)H(t))}{\epsilon} - (d-w\bar{L})\lambda^* H(t)\right)dt\right] + \lambda^*(x-(F+\eta)) \geq 0.$$

The decreasing property of $\tilde{u}(\cdot)$ endows us with $\tilde{u}(\lambda^* e^{\gamma t}D^\epsilon(t)H(t)) \leq \tilde{u}(\lambda^* e^{\gamma t}D^*(t)H(t))$. Applying Fatou's lemma, we have

$$\mathbb{E}\left[\int_0^{\tilde{\tau}} e^{-\gamma t}\tilde{u}'(\lambda^* e^{\gamma t}D^*(t)H(t))\lambda^* e^{\gamma t}H(t)dt\right] \geq \limsup_{\epsilon\downarrow 0}\mathbb{E}\left[\int_0^{\tilde{\tau}} e^{-\gamma t}\frac{\tilde{u}(\lambda^* e^{\gamma t}D^\epsilon(t)H(t)) - \tilde{u}(\lambda^* e^{\gamma t}D^*(t)H(t))}{\epsilon}dt\right].$$

Because of $c^*(t) + wl^*(t) = -\tilde{u}'(\lambda^* e^{\gamma t}D^*(t)H(t))$, we get

$$\mathbb{E}\left[\int_0^{\tilde{\tau}} H(t)(c^*(t) + wl^*(t) - w\bar{L} + d)dt\right] \leq x - (F+\eta). \tag{C.2}$$

Following the same technique, defining $D^\epsilon(t) \triangleq D^*(t) + \epsilon\mathbb{I}_{[0,\tau)}(t)$, we can obtain that

$$\mathbb{E}\left[\int_0^{\tau} H(t)(c^*(t) + wl^*(t) - w\bar{L} + d)dt\right] \leq x - (F+\eta). \tag{C.3}$$

We now follow the same argument as before, introducing a new process $\tilde{D}^\epsilon(t) \triangleq D^*(t) + \epsilon \in \mathcal{D}$, here ϵ is no longer required to be positive, but a sufficiently small real number. Defining a function

$$\begin{aligned}
\tilde{\mathfrak{L}}(D(t)) \triangleq& \mathbb{E}\left[\int_0^\tau e^{-\gamma t}\left(\tilde{u}(\lambda^* D(t)e^{\gamma t}H(t)) - (d-w\bar{L})\lambda^* e^{\gamma t}D(t)H(t)\right)dt + e^{-\gamma\tau}\tilde{U}(\lambda^* D(\tau)e^{\gamma\tau}H(\tau))\right]\\
&+ \lambda^*\mathbb{E}\left[\int_0^\tau (F+\eta)H(t)dD(t)\right] + \lambda^* xD(0),
\end{aligned}$$

we have $\tilde{\mathfrak{L}}(\tilde{D}^\epsilon(t)) \geq \tilde{\mathfrak{L}}(D^*(t))$, and

$$\begin{aligned}
\limsup_{\epsilon\downarrow 0}\frac{\tilde{\mathfrak{L}}(\tilde{D}^\epsilon(t)) - \tilde{\mathfrak{L}}(D^*(t))}{\epsilon} =& \limsup_{\epsilon\downarrow 0}\mathbb{E}\left[\int_0^\tau e^{-\gamma t}\frac{\tilde{u}(\lambda^* e^{\gamma t}\tilde{D}^\epsilon(t)H(t)) - \tilde{u}(\lambda^* e^{\gamma t}D^*(t)H(t))}{\epsilon}dt\right.\\
&\left.- \int_0^\tau (d-w\bar{L})\lambda^* H(t)dt + e^{-\gamma\tau}\frac{\tilde{U}(\lambda^* e^{\gamma\tau}\tilde{D}^\epsilon(\tau)H(\tau)) - \tilde{U}(\lambda^* e^{\gamma\tau}D^*(\tau)H(\tau))}{\epsilon}\right] + \lambda^* x \geq 0,
\end{aligned}$$

$$\liminf_{\epsilon\uparrow 0}\frac{\tilde{\mathfrak{L}}(\tilde{D}^{\epsilon}(t))-\tilde{\mathfrak{L}}(D^*(t))}{\epsilon}=\liminf_{\epsilon\uparrow 0}\mathbb{E}\left[\int_0^{\tau}e^{-\gamma t}\frac{\tilde{u}(\lambda^* e^{\gamma t}\tilde{D}^{\epsilon}(t)H(t))-\tilde{u}(\lambda^* e^{\gamma t}D^*(t)H(t))}{\epsilon}dt\right.$$
$$\left.-\int_0^{\tau}(d-w\bar{L})\lambda^* H(t)dt+e^{-\gamma\tau}\frac{\tilde{U}(\lambda^* e^{\gamma\tau}\tilde{D}^{\epsilon}(\tau)H(\tau))-\tilde{U}(\lambda^* e^{\gamma\tau}D^*(\tau)H(\tau))}{\epsilon}\right]+\lambda^* x\le 0.$$

Given the conditions

$$c^*(t)+wl^*(t)=-\tilde{u}'(\lambda^* e^{\gamma t}D^*(t)H(t)),\quad \text{and}\quad X^{x,c^*,\pi^*,l^*}(\tau)=-\tilde{U}'(\lambda^* e^{\gamma\tau}D^*(\tau)H(\tau)),$$

Fatou's lemma entails the following relations respectively:

$$\mathbb{E}\left[\int_0^{\tau}H(t)\left(c^*(t)+wl^*(t)-w\bar{L}+d\right)dt+H(\tau)X^{x,c^*,\pi^*,l^*}(\tau)\right]\le x,$$
$$\mathbb{E}\left[\int_0^{\tau}H(t)\left(c^*(t)+wl^*(t)-w\bar{L}+d\right)dt+H(\tau)X^{x,c^*,\pi^*,l^*}(\tau)\right]\ge x,$$

which lead to

$$\mathbb{E}\left[\int_0^{\tau}H(t)\left(c^*(t)+wl^*(t)-w\bar{L}+d\right)dt+H(\tau)X^{x,c^*,\pi^*,l^*}(\tau)\right]=x. \tag{C.4}$$

Subtracting Equation (C.4) from (C.2), we get

$$\mathbb{E}\left[\int_{\tilde{\tau}}^{\tau}H(t)(c(t)+wl(t)-w\bar{L}+d)dt+H(\tau)X^{x,c^*,\pi^*,l^*}(\tau)\right]\ge F+\eta,$$

which is equivalent to $\mathbb{E}\left[-\int_{\tilde{\tau}}^{\tau}H(t)(c(t)+wl(t)-w\bar{L}+d)dt-H(\tau)X^{x,c^*,\pi^*,l^*}(\tau)\right]\le -(F+\eta)$, for any stopping time $\tilde{\tau}\in\mathcal{S}$. Additionally, subtracting Equation (C.4) from (C.3), we obtain $\mathbb{E}[H(\tau)X^{x,c^*,\pi^*,l^*}(\tau)]\ge F+\eta$, which implies that $X^{x,c^*,\pi^*,l^*}(\tau)\ge F+\eta$ a.s.. Since $\tilde{\tau}$ can be any $\mathbb{F}$-stopping time in the set $\mathcal{S}$, there exists a portfolio strategy $\pi^*(t)$ that makes the corresponding wealth process satisfying $X^{x,c^*,\pi^*,l^*}(t)\ge F+\eta$, $\forall t\in[0,\tau]$, according to Lemma C.1.
(2) Then we turn to claim that $c^*(t)$ and $l^*(t)$ are the optimal consumption and leisure to the problem (P_τ). Taking an arbitrary control strategy $\{c(t),\pi(t),l(t)\}\in\mathcal{A}_\tau(x)$, the proof of Lemma C.1 guarantees that there exists a process $\zeta(t)$ satisfying

$$\int_0^t H(s)(c(s)+wl(s)-w\bar{L}+d)ds+H(t)X^{x,c,\pi,l}(t)=x+\int_0^t\zeta(s)dB(s),\quad \forall t\in[0,\tau]. \tag{C.5}$$

Since $X^{x,c,\pi,l}(t)\ge F+\eta$ a.s., $\forall t\in[0,\tau]$, we obtain the following inequality with any process $D(t)\in\mathcal{D}$,

$$\int_0^{\tau}\int_0^t H(s)(c(s)+wl(s)-w\bar{L}+d)ds dD(t)+\int_0^{\tau}(F+\eta)H(t)dD(t)\ge\int_0^{\tau}\left[x+\int_0^t\zeta(s)dB(s)\right]dD(t).$$

Since $D(t)$ is of bounded variation, we can implement the integration by parts and get

$$\int_0^{\tau}D(s)H(s)(c(s)+wl(s)-w\bar{L}+d)ds-\int_0^{\tau}D(s)\zeta(s)dB(s)\le D(0)x+\int_0^{\tau}(F+\eta)H(s)dD(s)$$
$$+D(\tau)\left[\int_0^{\tau}H(s)(c(s)+wl(s)-w\bar{L}+d)ds-x-\int_0^{\tau}\zeta(s)dB(s)\right].$$

Taking the expectation under the $\mathbb{P}$ measure on both sides and replacing Equation (C.5), we obtain

$$\mathbb{E}\left[\int_0^\tau D(s)H(s)(c(s)+wl(s)-w\bar{L}+d)ds\right]\le D(0)x-\mathbb{E}\big[D(\tau)H(\tau)X^{x,c,\pi,l}(\tau)\big]+\mathbb{E}\left[\int_0^\tau (F+\eta)H(s)dD(s)\right].$$

The above inequality keeps true for any admissible control strategy $\{c(t),\pi(t),l(t)\}$ and any non-negative, non-increasing process $D(t)$. Furthermore, we will show that the inequality changes into equality with the given $c^*(t)$, $l^*(t)$ and $D^*(t)$. We first define a new process

$$\bar{D}^\epsilon(t)\triangleq D^*(t)(1+\epsilon)\in\mathcal{D},$$

where ϵ is a small enough constant. Following the same argument in the first part proof, we have $\tilde{\mathfrak{L}}(\bar{D}^\epsilon(t))\ge\tilde{\mathfrak{L}}(D^*(t))$, and

$$\begin{aligned}\limsup_{\epsilon\downarrow 0}\frac{\tilde{\mathfrak{L}}(\bar{D}^\epsilon(t))-\tilde{\mathfrak{L}}(D^*(t))}{\epsilon}=&\limsup_{\epsilon\downarrow 0}\mathbb{E}\left[\int_0^\tau e^{-\gamma t}\frac{\tilde{u}(\lambda^*e^{\gamma t}D^*(t)(1+\epsilon)H(t))-\tilde{u}(\lambda^*e^{\gamma t}D^*(t)H(t))}{\epsilon}dt\right.\\&\left.-\int_0^\tau(d-w\bar{L})\lambda^*D^*(t)H(t)dt+e^{-\gamma\tau}\frac{\tilde{U}(\lambda^*e^{\gamma\tau}D^*(\tau)(1+\epsilon)H(\tau))-\tilde{U}(\lambda^*e^{\gamma\tau}D^*(\tau)H(\tau))}{\epsilon}\right]\\&+\lambda^*\mathbb{E}\left[\int_0^\tau(F+\eta)H(t)dD^*(t)\right]+\lambda^*xD^*(0)\ge 0,\end{aligned}$$

$$\begin{aligned}\liminf_{\epsilon\uparrow 0}\frac{\tilde{\mathfrak{L}}(\bar{D}^\epsilon(t))-\tilde{\mathfrak{L}}(D^*(t))}{\epsilon}=&\liminf_{\epsilon\uparrow 0}\mathbb{E}\left[\int_0^\tau e^{-\gamma t}\frac{\tilde{u}(\lambda^*e^{\gamma t}D^*(t)(1+\epsilon)H(t))-\tilde{u}(\lambda^*e^{\gamma t}D^*(t)H(t))}{\epsilon}dt\right.\\&\left.-\int_0^\tau(d-w\bar{L})\lambda^*D^*(t)H(t)dt+e^{-\gamma\tau}\frac{\tilde{U}(\lambda^*e^{\gamma\tau}D^*(\tau)(1+\epsilon)H(\tau))-\tilde{U}(\lambda^*e^{\gamma\tau}D^*(\tau)H(\tau))}{\epsilon}\right]\\&+\lambda^*\mathbb{E}\left[\int_0^\tau(F+\eta)H(t)dD^*(t)\right]+\lambda^*xD^*(0)\le 0.\end{aligned}$$

Applying Fatou's lemma, we obtain separately

$$\mathbb{E}\left[\int_0^\tau D^*(t)H(t)(c^*(t)+wl^*(t)-w\bar{L}+d)dt+D^*(\tau)H(\tau)X^{x,c^*,\pi^*,l^*}(\tau)\right]\le xD^*(0)+\mathbb{E}\left[\int_0^\tau(F+\eta)H(t)dD^*(t)\right],$$

$$\mathbb{E}\left[\int_0^\tau D^*(t)H(t)(c^*(t)+wl^*(t)-w\bar{L}+d)dt+D^*(\tau)H(\tau)X^{x,c^*,\pi^*,l^*}(\tau)\right]\ge xD^*(0)+\mathbb{E}\left[\int_0^\tau(F+\eta)H(t)dD^*(t)\right],$$

which give us

$$\mathbb{E}\left[\int_0^\tau D^*(t)H(t)(c^*(t)+wl^*(t)-w\bar{L}+d)dt+D^*(\tau)H(\tau)X^{x,c^*,\pi^*,l^*}(\tau)\right]=xD^*(0)+\mathbb{E}\left[\int_0^\tau(F+\eta)H(t)dD^*(t)\right].$$

Moreover, we define a new optimization problem named (P'_τ) as

$$\max_{c(t)\ge 0,l(t)\ge 0}\mathbb{E}\left[\int_0^\tau e^{-\gamma t}u(c(t),l(t))dt+e^{-\gamma\tau}U(X^{x,c,l}(\tau))\right],\qquad (P'_\tau)$$

subject to

$$\mathbb{E}\left[\int_0^\tau D^*(t)H(t)(c(t)+wl(t)-w\bar{L}+d)dt+D^*(\tau)H(\tau)X^{x,c,l}(\tau)\right]\le xD^*(0)+\mathbb{E}\left[\int_0^\tau(F+\eta)H(t)dD^*(t)\right].$$

We denote the optimal solutions of the above problem as $\tilde{c}^*(t)$ and $\tilde{l}^*(t)$.The Lagrange method endows us

$$\tilde{c}^*(t) + w\tilde{l}^*(t) = -\tilde{u}'(\tilde{\lambda} e^{\gamma t} D^*(t)H(t)), \quad X^{x,\tilde{c}^*,\tilde{l}^*}(\tau) = -\tilde{U}'(\tilde{\lambda} e^{\gamma \tau} D^*(\tau)H(\tau)),$$

where $\tilde{\lambda} > 0$ is the Lagrange multiplier. The constraint of Problem (P'_τ) takes equality when $\tilde{\lambda} = \lambda^*$. Then, the condition $c^*(t) + wl^*(t) = -\tilde{u}'(\lambda^* e^{\gamma t} D^*(t)H(t))$ implies that $c^*(t)$ and $l^*(t)$ are the optimal control policies of the problem (P'_τ). Moreover, since the maximum utility of primal problem (P_τ) is upper bounded by the maximum utility of (P'_τ), we can conclude that $c^*(t)$ and $l^*(t)$ are also the optimal consumption and leisure solution to the problem (P_τ).

C.3 Proof of Lemma 4.1

Referring to (Oksendal, 2013, Section 10.3, Example 10.3.1), the notations here almost coincide with the ones there. Let us first introduce two new functions:

$$g(y) \triangleq g(t,z) = e^{-\gamma t}\tilde{U}(z), \quad \text{and} \quad G(t,z,\bar{w}) \triangleq g(t,z) + \bar{w} = e^{-\gamma t}\tilde{U}(z) + \bar{w}.$$

Defining an operator $\mathcal{A}_P G \triangleq \frac{\partial G}{\partial t} + (\gamma - r) z \frac{\partial G}{\partial z} + \frac{\theta^2}{2} z^2 \frac{\partial^2 G}{\partial z^2} + e^{-\gamma t}\tilde{u}(z) - e^{-\gamma t}(d - w\bar{L})z$, the continuous region of the corresponding optimal stopping time problem is expressed as

$$\Omega_1 = \{(t,z,\bar{w}) : \mathcal{A}_P G(t,z,\bar{w}) > 0\}.$$

Since

$$\mathcal{A}_P G(t,z,\bar{w}) = -\gamma e^{-\gamma t}\tilde{U}(z) + (\gamma - r) z e^{-\gamma t}\tilde{U}'(z) + \frac{\theta^2}{2} z^2 e^{-\gamma t}\tilde{U}''(z) + e^{-\gamma t}\tilde{u}(z) - e^{-\gamma t}(d - w\bar{L})z,$$

defining a new function $h(z) = -\gamma\tilde{U}(z) + (\gamma - r)z\tilde{U}'(z) + \frac{\theta^2}{2} z^2 \tilde{U}''(z) + \tilde{u}(z) - (d - w\bar{L})z$, the continuous region can be rewritten as $\Omega_1 = \{z : h(z) > 0\}$. Because $\tilde{U}(z)$ takes the piecewise form of

$$\tilde{U}(z) = \begin{cases} v_{PB}\left(\frac{z}{\alpha}\right) - Fz, & 0 < z < \alpha\hat{z}_{PB}, \\ v_{PB}(\hat{z}_{PB}) - Fz, & z \geq \alpha\hat{z}_{PB}, \end{cases}$$

we can rewrite $h(z)$ as

$$\begin{aligned} h(z) &= \begin{cases} -\gamma v_{PB}\left(\frac{z}{\alpha}\right) + (\gamma - r)\frac{z}{\alpha} v'_{PB}\left(\frac{z}{\alpha}\right) + \frac{\theta^2}{2}\frac{z^2}{\alpha^2} v''_{PB}\left(\frac{z}{\alpha}\right) + rFz + \tilde{u}(z) - (d - w\bar{L})z, & 0 < z < \alpha\hat{z}_{PB}, \\ -v_{PB}(\hat{z}_{PB}) + \tilde{u}(z) + (rF - d + w\bar{L})z, & z \geq \alpha\hat{z}_{PB}, \end{cases} \\ &= \begin{cases} \tilde{u}(z) - \tilde{u}\left(\frac{z}{\alpha}\right) + (rF - d + w\bar{L} - \frac{w\bar{L}}{\alpha})z, & 0 < z < \alpha\hat{z}_{PB}, \\ -v_{PB}(\hat{z}_{PB}) + \tilde{u}(z) + (rF - d + w\bar{L})z, & z \geq \alpha\hat{z}_{PB}, \end{cases} \end{aligned}$$

the last equality comes from Condition (B.2) on the interval $0 < z < \alpha\hat{z}_{PB}$. From Condition (4.5), it can be observed that the function $h(z)$ only takes the form

$$h(z) = \tilde{u}(z) - \tilde{u}\left(\frac{z}{\alpha}\right) + (rF - d + w\bar{L} - \frac{w\bar{L}}{\alpha})z,$$

on the considering interval $0 < z < \hat{z}$. We first extend the domain of $h(z)$ to the whole positive real line, and discover the existence and uniqueness of its zero $\bar{z}$, then discuss the magnitude between $\bar{z}$

and $\hat{z}$. The decreasing property of $\tilde{u}(z)$ leads to $\tilde{u}(z) - \tilde{u}\left(\frac{z}{\alpha}\right) > 0$; hence, a necessary condition to ensure that there is at least one zero point is put forward as $rF - d + w\bar{L} - \frac{w\bar{L}}{\alpha} < 0$. Afterwards, for the sake of determining the curvature of $h(z)$, we take the second-order derivative and obtain

$$h''(z) = \tilde{u}''(z) - \frac{1}{\alpha^2}\tilde{u}''\left(\frac{z}{\alpha}\right),$$

therefore we focus on the sign of function $\tilde{u}''(z) - \frac{1}{\alpha^2}\tilde{u}''\left(\frac{z}{\alpha}\right)$. Since $\tilde{u}(z)$ is strictly decreasing and convex on $(0, \infty)$ and adopts the piecewise form, we discover the sign of $\tilde{u}''(z) - \frac{1}{\alpha^2}\tilde{u}''\left(\frac{z}{\alpha}\right)$ on three different intervals: $z < \alpha\tilde{y}$, $\alpha\tilde{y} \le z < \tilde{y}$ and $z \ge \tilde{y}$.

- On $z < \alpha\tilde{y}$: from $\frac{\delta(1-k)}{1-\delta(1-k)} < 0$, $0 < \alpha < 1$ and $A_1 = \frac{1-\delta+\delta k}{\delta(1-k)} L^{-\frac{(1-k)(1-\delta)}{\delta(1-k)-1}} < 0$, we get

$$\tilde{u}''(z) - \frac{1}{\alpha^2}\tilde{u}''\left(\frac{z}{\alpha}\right) = \frac{\delta(1-k)}{(\delta(1-k)-1)^2} A_1 z^{\frac{2-\delta(1-k)}{\delta(1-k)-1}} \left(1 - \alpha^{\frac{\delta(1-k)}{1-\delta(1-k)}}\right) < 0.$$

- On $\alpha\tilde{y} \le z < \tilde{y}$: the function $\tilde{u}''(z) - \frac{1}{\alpha^2}\tilde{u}''\left(\frac{z}{\alpha}\right)$ is rewritten as

$$\tilde{u}''(z) - \frac{1}{\alpha^2}\tilde{u}''\left(\frac{z}{\alpha}\right) = \frac{1}{z^2}\left[\frac{\delta(1-k)}{(\delta(1-k)-1)^2} A_1 z^{\frac{\delta(1-k)}{\delta(1-k)-1}} - \frac{1-k}{k^2} A_2 z^{-\frac{1-k}{k}} \alpha^{\frac{1-k}{k}}\right].$$

  The first term inside the square bracket has the following inequality relationship

$$\frac{\delta(1-k)}{(\delta(1-k)-1)^2} A_1 z^{\frac{\delta(1-k)}{\delta(1-k)-1}} < \frac{\delta(1-k)}{(\delta(1-k)-1)^2} A_1 \tilde{y}^{\frac{\delta(1-k)}{\delta(1-k)-1}} = \frac{1}{1-\delta(1-k)} L^{1-k} \left(\frac{1-\delta}{\delta w}\right)^{-\delta(1-k)},$$

  as for the second term, we have

$$-\frac{1-k}{k^2} A_2 z^{-\frac{1-k}{k}} \alpha^{\frac{1-k}{k}} \le -\frac{1-k}{k^2} A_2 (\alpha\tilde{y})^{-\frac{1-k}{k}} \alpha^{\frac{1-k}{k}} = -\frac{L^{1-k}}{\delta}\left(\frac{1-\delta}{\delta w}\right)^{-\delta(1-k)}.$$

  Then we can determine the sign of $\tilde{u}''(z) - \frac{1}{\alpha^2}\tilde{u}''\left(\frac{z}{\alpha}\right)$ as

$$\begin{aligned}\tilde{u}''(z) - \frac{1}{\alpha^2}\tilde{u}''\left(\frac{z}{\alpha}\right) &< \frac{1}{z^2}\left[\frac{1}{1-\delta(1-k)} L^{1-k}\left(\frac{1-\delta}{\delta w}\right)^{-\delta(1-k)} - \frac{L^{1-k}}{\delta}\left(\frac{1-\delta}{\delta w}\right)^{-\delta(1-k)}\right] \\ &= \frac{1}{z^2}\left[\left(\frac{1-\delta}{\delta w}\right)^{-\delta(1-k)} L^{1-k}\left(\frac{\delta - 1 + \delta(1-k)}{(1-\delta(1-k))\delta}\right)\right] < 0.\end{aligned}$$

- On $z \ge \tilde{y}$: The conditions $\frac{1-k}{k} < 0$, $0 < \alpha < 1$ and $A_2 = \frac{k}{\delta(1-k)}\left(\frac{1-\delta}{\delta w}\right)^{\frac{(1-k)(1-\delta)}{k}} < 0$ endows us

$$\tilde{u}''(z) - \frac{1}{\alpha^2}\tilde{u}''\left(\frac{z}{\alpha}\right) = \frac{1-k}{k^2} A_2 z^{-\frac{1}{k}-1}\left(1 - \alpha^{\frac{1-k}{k}}\right) < 0.$$

Hence, we can summarize that $h''(z) < 0$, which means the function $h(z)$ is strictly concave for $z > 0$. Additionally, combining with the subsequent facts

$$\lim_{z\downarrow 0} h(z) = \lim_{z\downarrow 0} A_1 z^{\frac{\delta(1-k)}{\delta(1-k)-1}}\left(1 - \alpha^{\frac{\delta(1-k)}{1-\delta(1-k)}}\right) + wLz\left(\frac{1}{\alpha} - 1\right) + \left(rF - d + w\bar{L} - \frac{w\bar{L}}{\alpha}\right) z = 0,$$

$$\lim_{z\downarrow 0} h'(z) = \lim_{z\downarrow 0} \frac{\delta(1-k)}{\delta(1-k)-1} A_1 z^{\frac{1}{\delta(1-k)-1}} \left(1-\alpha^{\frac{\delta(1-k)}{1-\delta(1-k)}}\right) + rF - d + w(\bar{L}-L)\left(1-\frac{1}{\alpha}\right) = \infty,$$

$$\lim_{z\to\infty} h(z) = \lim_{z\to\infty} A_2 z^{-\frac{1-k}{k}} \left(1-\alpha^{\frac{1-k}{k}}\right) + \left(rF - d + w\bar{L} - \frac{w\bar{L}}{\alpha}\right) z = -\infty,$$

we can conclude that there exists a unique $\bar{z}$ such that $\{z>0 : h(z)>0\} = \{0<z<\bar{z}\}$. Moreover, solutions of the primal optimization problem are discussed in two different cases, $\bar{z}<\hat{z}$ and $\bar{z}\geq\hat{z}$.

C.4 Proof of Lemma 4.2

Referring to (Jeanblanc et al., 2004, Appendix, A.2), we make use of the scale function to calculate the probability of the stopping time. Related to the diffusion process of $Z(t)$, the scale function is

$$s(z) = \int_a^z e^{-2\int_a^y \frac{f(x)}{g^2(x)}dx} dy,$$

where $f(x) = (\gamma - r)x$, $g(x) = -\theta x$ are the drift and diffusion coefficients of $dZ(t)$ and a is an arbitrary constant located in $(0, \bar{z})$. Then we obtain

$$\mathbb{P}(\tau_{\bar{z}} < \tau_0) = \frac{s(z)-s(0)}{s(\bar{z})-s(0)},$$

with $s(0) = \lim_{b\to 0^+} \int_a^b e^{-2\int_a^y \frac{\gamma-r}{\theta^2 x}dx} dy$. It follows that in order to prove that the above probability is one instead of depending on the initial value z, it suffices to show that $s(0) = -\infty$. Since

$$s(0) = \lim_{b\to 0^+} \int_a^b e^{-2\int_a^y \frac{\gamma-r}{\theta^2 x}dx} dy = \lim_{b\to 0^+} \left(\frac{a^{\frac{2(\gamma-r)}{\theta^2}}}{1-\frac{2(\gamma-r)}{\theta^2}} b^{1-\frac{2(\gamma-r)}{\theta^2}} - \frac{a}{1-\frac{2(\gamma-r)}{\theta^2}} \right),$$

a sufficient condition to make $s(0) = -\infty$ is $1 - \frac{2(\gamma-r)}{\theta^2} < 0$, which is equal to $\gamma > r + \frac{\theta^2}{2}$. Moreover, following the same argument, it can be easily proven that $\mathbb{P}(\tau_0 < \infty) = 1$ with the condition (4.7), which also gives us $\mathbb{P}(\tau_{\bar{z}} < \infty) = 1$.

C.5 Solutions of Variational Inequalities (4.8) and (4.9)

Case 1. $0 < \bar{z} < \hat{z} < \alpha\tilde{y} \leq \alpha\hat{z}_{PB}$**:** The differential equation generated from Condition $(V3)$ in the system (4.8) takes the solution as

$$v(z) = B_1 z^{n_1} + B_2 z^{n_2} + \frac{A_1}{\Gamma_1} z^{\frac{\delta(1-k)}{\delta(1-k)-1}} + \frac{w(\bar{L}-L)-d}{r} z, \qquad 0<z<\bar{z}.$$

Since $n_1 < 0$, the term z^{n_1} will suffer the explosion as z goes to 0. Therefore, we set the coefficient $B_1 = 0$ to meet the boundedness assumption of $v(z)$. Forward, the smooth fit condition $v(\bar{z}) = \tilde{U}(\bar{z})$ leads to the subsequent two-equation system to resolve the parameters B_2 and $\bar{z}$.

- $\mathcal{C}^0$ condition at $z = \bar{z}$

$$B_2\bar{z}^{n_2} + \frac{A_1}{\Gamma_1}\bar{z}^{\frac{\delta(1-k)}{\delta(1-k)-1}} + \frac{w(\bar{L}-L)-d}{r}\bar{z} = B_{21,PB}\left(\frac{\bar{z}}{\alpha}\right)^{n_2} + \frac{A_1}{\Gamma_1}\left(\frac{\bar{z}}{\alpha}\right)^{\frac{\delta(1-k)}{\delta(1-k)-1}} + \left(\frac{w(\bar{L}-L)}{r\alpha} - F\right)\bar{z}. \quad \text{(C.6)}$$

- $\mathcal{C}^1$ condition at $z = \bar{z}$

$$n_2 B_2 \bar{z}^{n_2-1} + \frac{\delta(1-k)}{\delta(1-k)-1}\frac{A_1}{\Gamma_1}\bar{z}^{\frac{1}{\delta(1-k)-1}} + \frac{w(\bar{L}-L)-d}{r}$$
$$= n_2 \frac{B_{21,PB}}{\alpha}\left(\frac{\bar{z}}{\alpha}\right)^{n_2-1} + \frac{\delta(1-k)}{\delta(1-k)-1}\frac{A_1}{\Gamma_1\alpha}\left(\frac{\bar{z}}{\alpha}\right)^{\frac{1}{\delta(1-k)-1}} + \left(\frac{w(\bar{L}-L)}{r\alpha} - F\right). \tag{C.7}$$

Besides, the condition $\tilde{U}'(\hat{z}) = -(F+\eta)$ generates the following equation

$$\tilde{U}(\hat{z}) = n_2 \frac{B_{21,PB}}{\alpha}\left(\frac{\hat{z}}{\alpha}\right)^{n_2-1} + \frac{\delta(1-k)}{\delta(1-k)-1}\frac{A_1}{\Gamma_1\alpha}\left(\frac{\hat{z}}{\alpha}\right)^{\frac{1}{\delta(1-k)-1}} + \frac{w(\bar{L}-L)}{r\alpha} = -\eta,$$

which endows us the value of $\hat{z}$.

Case 2. $0 < \bar{z} < \alpha\tilde{y} \le \hat{z} \le \alpha\hat{z}_{PB}$**:** The Condition $(V3)$ from (4.8) leads to the following differential equation

$$-\gamma v(z) + (\gamma - r)zv'(z) + \frac{1}{2}\theta^2 z^2 v''(z) + \tilde{u}(z) - (d - w\bar{L})z = 0, \quad 0 < z < \bar{z},$$

since the condition $\bar{z} < \tilde{y}$ remains unchanged, the above equation keeps the same compared with the previous sections, hence, takes the identical solution as

$$v(z) = B_2 z^{n_2} + \frac{A_1}{\Gamma_1} z^{\frac{\delta(1-k)}{\delta(1-k)-1}} + \frac{w(\bar{L}-L)-d}{r}z, \qquad 0 < z < \bar{z}.$$

Furthermore, because the condition $\bar{z} < \alpha\tilde{y}$ also coincides with Case 1, we have the same smooth fit condition $v(\bar{z}) = \tilde{U}(\bar{z})$, which results in the identical values of parameters B_2 and $\bar{z}$. The difference from the previous case occurs at the boundary $\hat{z}$. Because of $\alpha\tilde{y} \le \hat{z} \le \alpha\hat{z}_{PB}$, the function $\tilde{U}(z)$ adopts a different form at the point $\hat{z}$; furthermore, $(V1)$ and $(V6)$ in the system (4.8) produces a different equation, that is,

$$n_1 \frac{B_{12,PB}}{\alpha}\left(\frac{\hat{z}}{\alpha}\right)^{n_1-1} + n_2 \frac{B_{22,PB}}{\alpha}\left(\frac{\hat{z}}{\alpha}\right)^{n_2-1} - \frac{1-k}{k}\frac{A_2}{\Gamma_2\alpha}\left(\frac{\hat{z}}{\alpha}\right)^{-\frac{1}{k}} + \frac{w\bar{L}}{r\alpha} = -\eta, \tag{C.8}$$

to get the value of $\hat{z}$.

Case 3. $0 < \alpha\tilde{y} < \bar{z} < \hat{z} \le \alpha\hat{z}_{PB}$ & $\bar{z} < \tilde{y}$**:** Under the same condition $\bar{z} < \tilde{y}$ with the previous cases, the differential equation generating from $(V3)$ of (4.8) follows the identical solution,

$$v(z) = B_2 z^{n_2} + \frac{A_1}{\Gamma_1} z^{\frac{\delta(1-k)}{\delta(1-k)-1}} + \frac{w(\bar{L}-L)-d}{r}z, \qquad 0 < z < \bar{z}.$$

Then the smooth fit condition $v(\bar{z}) = \tilde{U}(\bar{z})$ enables us to determine the values of parameters B_2 and $\bar{z}$ with the following two-equation system:

- $\mathcal{C}^0$ condition at $z = \bar{z}$

$$B_2\bar{z}^{n_2}+\frac{A_1}{\Gamma_1}\bar{z}^{\frac{\delta(1-k)}{\delta(1-k)-1}}+\frac{w(\bar{L}-L)-d}{r}\bar{z}=B_{12,PB}\left(\frac{\bar{z}}{\alpha}\right)^{n_1}+B_{22,PB}\left(\frac{\bar{z}}{\alpha}\right)^{n_2}+\frac{A_2}{\Gamma_2}\left(\frac{\bar{z}}{\alpha}\right)^{-\frac{1-k}{k}}+\left(\frac{w\bar{L}}{r\alpha}-F\right)\bar{z};$$

- $\mathcal{C}^1$ condition at $z = \bar{z}$

$$n_2B_2\bar{z}^{n_2-1}+\frac{\delta(1-k)}{\delta(1-k)-1}\frac{A_1}{\Gamma_1}\bar{z}^{\frac{1}{\delta(1-k)-1}}+\frac{w(\bar{L}-L)-d}{r}=$$
$$n_1\frac{B_{12,PB}}{\alpha}\left(\frac{\bar{z}}{\alpha}\right)^{n_1-1}+n_2\frac{B_{22,PB}}{\alpha}\left(\frac{\bar{z}}{\alpha}\right)^{n_2-1}-\frac{1-}{k}\frac{A_2}{\Gamma_2\alpha}\left(\frac{\bar{z}}{\alpha}\right)^{-\frac{1}{k}}+\left(\frac{w\bar{L}}{r\alpha}-F\right).$$

Besides, from Condition $(V1)$ and $(V6)$ of (4.8), we have the smooth fit condition $\tilde{U}'(\hat{z}) = -(F+\eta)$. Combining with the prerequisite $\alpha\tilde{y} \le \hat{z} \le \alpha\hat{z}_{PB}$, we have the subsequent equation,

$$n_1\frac{B_{12,PB}}{\alpha}\left(\frac{\hat{z}}{\alpha}\right)^{n_1-1}+n_2\frac{B_{22,PB}}{\alpha}\left(\frac{\hat{z}}{\alpha}\right)^{n_2-1}-\frac{1-k}{k}\frac{A_2}{\Gamma_2\alpha}\left(\frac{\hat{z}}{\alpha}\right)^{-\frac{1}{k}}+\frac{w\bar{L}}{r\alpha}=-\eta,$$

which gives us $\hat{z}$.

Case 4. $0 < \bar{z} < \hat{z} \le \alpha\hat{z}_{PB} < \alpha\tilde{y} < \tilde{y}$**:** Same with the previous cases, Condition $(V3)$ of (4.8) forces the function $v(z)$ to take the solution as

$$v(z) = B_2z^{n_2}+\frac{A_1}{\Gamma_1}z^{\frac{\delta(1-k)}{\delta(1-k)-1}}+\frac{w(\bar{L}-L)-d}{r}z, \qquad 0<z<\bar{z}.$$

Then, considering the smooth fit condition $v(\bar{z}) = \tilde{U}(\bar{z})$, a two-equation system is established to resolve the parameters B_2 and $\bar{z}$:

- $\mathcal{C}^0$ condition at $z = \bar{z}$

$$B_2\bar{z}^{n_2}+\frac{A_1}{\Gamma_1}\bar{z}^{\frac{\delta(1-k)}{\delta(1-k)-1}}+\frac{w(\bar{L}-L)-d}{r}\bar{z}=B_{2,PB}\left(\frac{\bar{z}}{\alpha}\right)^{n_2}+\frac{A_1}{\Gamma_1}\left(\frac{\bar{z}}{\alpha}\right)^{\frac{\delta(1-k)}{\delta(1-k)-1}}+\left(\frac{w(\bar{L}-L)}{r\alpha}-F\right)\bar{z};$$

- $\mathcal{C}^1$ condition at $z = \bar{z}$

$$n_2B_2\bar{z}^{n_2-1}+\frac{\delta(1-k)}{\delta(1-k)-1}\frac{A_1}{\Gamma_1}\bar{z}^{\frac{1}{\delta(1-k)-1}}+\frac{w(\bar{L}-L)-d}{r}$$
$$=n_2\frac{B_{2,PB}}{\alpha}\left(\frac{\bar{z}}{\alpha}\right)^{n_2-1}+\frac{\delta(1-k)}{\delta(1-k)-1}\frac{A_1}{\Gamma_1\alpha}\left(\frac{\bar{z}}{\alpha}\right)^{\frac{1}{\delta(1-k)-1}}+\left(\frac{w(\bar{L}-L)}{r\alpha}-F\right).$$

Combining the condition $\tilde{U}'(\hat{z}) = -(F+\eta)$ with the prerequisite $\hat{z} \le \hat{z}_{PB}$, we get the following equation

$$n_2\frac{B_{2,PB}}{\alpha}\left(\frac{\hat{z}}{\alpha}\right)^{n_2-1}+\frac{\delta(1-k)}{\delta(1-k)-1}\frac{A_1}{\Gamma_1\alpha}\left(\frac{\hat{z}}{\alpha}\right)^{\frac{1}{\delta(1-k)-1}}+\frac{w(\bar{L}-L)}{r\alpha}=-\eta,$$

which enables us to obtain $\hat{z}$.

Case 5. $0 < \alpha\tilde{y} < \tilde{y} \le \bar{z} < \hat{z} < \alpha\hat{z}_{PB}$**:** We begin with Condition $(V3)$ of (4.8), which leads to the subsequent differential equation on the interval $0 < z < \bar{z}$,

$$-\gamma v(z) + (\gamma - r)zv'(z) + \frac{1}{2}\theta^2 z^2 v''(z) + \tilde{u}(z) - (d - w\bar{L})z = 0.$$

Since $\bar{z} > \tilde{y}$, Lemma 2.1 shows that the function $\tilde{u}(z)$ takes two different forms on the corresponding interval with $\tilde{y}$ as the separating threshold. Hence, the solution of the above differential equation inherits this piecewise property and has the following resolution,

$$v(z) = \begin{cases} B_{11}z^{n_1} + B_{21}z^{n_2} + \frac{A_1}{\Gamma_1}z^{\frac{\delta(1-k)}{\delta(1-k)-1}} + \frac{w(\bar{L}-L)-d}{r}z, & 0 < z < \tilde{y}, \\ B_{12}z^{n_1} + B_{22}z^{n_2} + \frac{A_2}{\Gamma_2}z^{-\frac{1-k}{k}} + \frac{w\bar{L}-d}{r}z, & \tilde{y} \le z < \bar{z}. \end{cases}$$

To meet the boundedness assumption of $v(z)$, we set $B_{11} = 0$ to avoid the explosion of term z^{n_1} as z goes to 0. Then, the same argument with the previous cases, we need to construct a four-equation system, which resorts to the smooth conditions at the point $\tilde{y}$ and $\bar{z}$, to determine the boundary $\bar{z}$, and the coefficient of function $v(z)$, i.e., B_{21}, B_{12}, B_{22}:

- $\mathcal{C}^0$ condition at $z = \bar{z}$

$$B_{12}\bar{z}^{n_1} + B_{22}\bar{z}^{n_2} + \frac{A_2}{\Gamma_2}\bar{z}^{-\frac{1-k}{k}} + \frac{w\bar{L}-d}{r}\bar{z} = B_{12,PB}\left(\frac{\bar{z}}{\alpha}\right)^{n_1} + B_{22,PB}\left(\frac{\bar{z}}{\alpha}\right)^{n_2} + \frac{A_2}{\Gamma_2}\left(\frac{\bar{z}}{\alpha}\right)^{-\frac{1-k}{k}} + \left(\frac{w\bar{L}}{r\alpha} - F\right)\bar{z};$$

- $\mathcal{C}^1$ condition at $z = \bar{z}$

$$n_1 B_{12}\bar{z}^{n_1-1} + n_2 B_{22}\bar{z}^{n_2-1} - \frac{1-k}{k}\frac{A_2}{\Gamma_2}\bar{z}^{-\frac{1}{k}} + \frac{w\bar{L}-d}{r} = n_1\frac{B_{12,PB}}{\alpha}\left(\frac{\bar{z}}{\alpha}\right)^{n_1-1} + n_2\frac{B_{22,PB}}{\alpha}\left(\frac{\bar{z}}{\alpha}\right)^{n_2-1} - \frac{1-k}{k}\frac{A_2}{\Gamma_2\alpha}\left(\frac{\bar{z}}{\alpha}\right)^{-\frac{1}{k}} + \frac{w\bar{L}}{r\alpha} - F;$$

- $\mathcal{C}^0$ condition at $z = \tilde{y}$

$$B_{21}\tilde{y}^{n_2} + \frac{A_1}{\Gamma_1}\tilde{y}^{\frac{\delta(1-k)}{\delta(1-k)-1}} - \frac{wL}{r}\tilde{y} = B_{12}\tilde{y}^{n_1} + B_{22}\tilde{y}^{n_2} + \frac{A_2}{\Gamma_2}\tilde{y}^{-\frac{1-k}{k}};$$

- $\mathcal{C}^1$ condition at $z = \tilde{y}$

$$n_2 B_{21}\tilde{y}^{n_2-1} + \frac{\delta(1-k)}{\delta(1-k)-1}\frac{A_1}{\Gamma_1}\tilde{y}^{\frac{1}{\delta(1-k)-1}} - \frac{wL}{r} = n_1 B_{12}\tilde{y}^{n_1-1} + n_2 B_{22}\tilde{y}^{n_2-1} - \frac{1-k}{k}\frac{A_2}{\Gamma_2}\tilde{y}^{-\frac{1}{k}}.$$

Meanwhile, considering the prerequisite $\alpha\tilde{y} < \hat{z} < \alpha\hat{z}_{PB}$ and the smooth fit condition $\tilde{U}'(\hat{z}) = -(F+\eta)$, we have

$$n_1\frac{B_{12,PB}}{\alpha}\left(\frac{\hat{z}}{\alpha}\right)^{n_1-1} + n_2\frac{B_{22,PB}}{\alpha}\left(\frac{\hat{z}}{\alpha}\right)^{n_2-1} - \frac{1-k}{k}\frac{A_2}{\Gamma_2\alpha}\left(\frac{\hat{z}}{\alpha}\right)^{-\frac{1}{k}} + \frac{w\bar{L}}{r\alpha} = -\eta,$$

which gives us the value of $\hat{z}$.

Case 6. $0 < \tilde{y} \le \hat{z}$ & $\bar{z} \ge \hat{z}$**:** The Condition $(V3)$ of (4.9) endows us a partial differential equation with the solution

$$v(z) = \begin{cases} B_{11}z^{n_1} + B_{21}z^{n_2} + \frac{A_1}{\Gamma_1}z^{\frac{\delta(1-k)}{\delta(1-k)-1}} + \frac{w(\bar{L}-L)-d}{r}z, & 0 < z < \tilde{y}, \\ B_{12}z^{n_1} + B_{22}z^{n_2} + \frac{A_2}{\Gamma_2}z^{-\frac{1-k}{k}} + \frac{w\bar{L}-d}{r}z, & \tilde{y} \le z < \hat{z}. \end{cases}$$

Due to the same considerations as before, we set $B_{11} = 0$ to meet the boundedness of $v(z)$. Then, with the smooth fit conditions at $\hat{z}$ and $\tilde{y}$, we can construct a four-equation system to determine the values of parameters B_{21}, B_{12}, B_{22} and $\hat{z}$:

- $\mathcal{C}^0$ condition at $z = \tilde{y}$

$$B_{21}\tilde{y}^{n_2} + \frac{A_1}{\Gamma_1}\tilde{y}^{\frac{\delta(1-k)}{\delta(1-k)-1}} - \frac{wL}{r}\tilde{y} = B_{12}\tilde{y}^{n_1} + B_{22}\tilde{y}^{n_2} + \frac{A_2}{\Gamma_2}\tilde{y}^{-\frac{1-k}{k}};$$

- $\mathcal{C}^1$ condition at $z = \tilde{y}$

$$n_2B_{21}\tilde{y}^{n_2-1} + \frac{\delta(1-k)}{\delta(1-k)-1}\frac{A_1}{\Gamma_1}\tilde{y}^{\frac{1}{\delta(1-k)-1}} - \frac{wL}{r} = n_1B_{12}\tilde{y}^{n_1-1} + n_2B_{22}\tilde{y}^{n_2-1} - \frac{1-k}{k}\frac{A_2}{\Gamma_2}\tilde{y}^{-\frac{1}{k}};$$

- $\mathcal{C}^1$ condition at $z = \hat{z}$

$$n_1B_{12}\hat{z}^{n_1-1} + n_2B_{22}\hat{z}^{n_2-1} - \frac{1-k}{k}\frac{A_2}{\Gamma_2}\hat{z}^{-\frac{1}{k}} + \frac{w\bar{L}-d}{r} + F + \eta = 0;$$

- $\mathcal{C}^2$ condition at $z = \hat{z}$

$$n_1(n_1-1)B_{12}\hat{z}^{n_1-2} + n_2(n_2-1)B_{22}\hat{z}^{n_2-2} + \frac{1-k}{k^2}\frac{A_2}{\Gamma_2}\hat{z}^{-\frac{1+k}{k}} = 0.$$

Case 7. $0 < \hat{z} < \tilde{y}$ & $\bar{z} \ge \hat{z}$**:** The prerequisite $\hat{z} < \tilde{y}$ makes the function $\tilde{u}(z)$ of the form $A_1z^{\frac{\delta(1-k)}{\delta(1-k)-1}} - wLz$. Then Condition $(V3)$ in (4.9) has the solution

$$v(z) = B_1z^{n_1} + B_2z^{n_2} + \frac{A_1}{\Gamma_1}z^{\frac{\delta(1-k)}{\delta(1-k)-1}} + \frac{w(\bar{L}-L)-d}{r}z, \quad 0 < z < \hat{z}.$$

We set $B_1 = 0$ for avoiding the explosion of the term $B_1z^{n_1}$ when z goes to 0. Then a two-equation system is created to solve the value of parameters B_2 and $\hat{z}$ explicitly:

- $\mathcal{C}^1$ condition at $z = \hat{z}$

$$n_2B_2\hat{z}^{n_2-1} + \frac{\delta(1-k)}{\delta(1-k)-1}\frac{A_1}{\Gamma_1}\hat{z}^{\frac{1}{\delta(1-k)-1}} + \frac{w(\bar{L}-L)-d}{r} + F + \eta = 0;$$

- $\mathcal{C}^2$ condition at $z = \hat{z}$

$$n_2(n_2-1)B_2\hat{z}^{n_2-2} + \frac{\delta(1-k)}{(\delta(1-k)-1)^2}\frac{A_1}{\Gamma_1}\hat{z}^{\frac{2-\delta(1-k)}{\delta(1-k)-1}} = 0.$$

D No Optimal Bankruptcy Problem

To study the influence of introducing the bankruptcy option, we also solve a pure optimal control problem without optimal stopping, which is defined subsequently as

$$V_{nob}(x) = \sup_{\{c_{nob}(t),\pi_{nob}(t),l_{nob}(t)\}_{t\geq 0}\in\mathcal{A}_{nob}(x)} \mathbb{E}\left[\int_0^\infty e^{-\gamma t}u(c_{nob}(t), l_{nob}(t))dt\right].$$

The subscript $_{nob}$ indicates that the considering variables and functions are concerned with the no bankruptcy option model. Moreover, the admissible control set $\mathcal{A}_{nob}(x)$ is almost consistent with the definition of $\mathcal{A}_{PB}(x)$ except the liquidity condition. $\mathcal{A}_{nob}(x)$ adopts "$X_{nob}(t) \geq F + \eta$, a.s., $\forall t \geq 0$" instead of "$X(t) \geq 0$, a.s., $\forall t \geq 0$". Then we provide the budget and liquidity constraints as:

$$\begin{cases} \text{Budget Constraint:} & \mathbb{E}\left[\int_0^\infty H(t)(c_{nob}(t) + d + wl_{nob}(t) - w\bar{L})dt\right] \leq x, \\ \text{Liquidity Constraint:} & \mathbb{E}\left[\int_t^\infty \frac{H(s)}{H(t)}(c_{nob}(s) + d + wl_{nob}(s) - w\bar{L})ds \Big| \mathcal{F}_t\right] \geq F + \eta. \end{cases}$$

Following the same argument with the post-bankruptcy part, we can solve the optimal control problem with two different cases:

- Case 1. $0 < \tilde{y} \leq \hat{z}_{nob}$: the optimal consumption-portfolio-leisure strategy is

$$c^*_{nob}(t) = \begin{cases} L^{-\frac{(1-k)(1-\delta)}{\delta(1-k)-1}}(Z^*_{nob}(t))^{\frac{1}{\delta(1-k)-1}}, & 0 < Z^*_{nob}(t) < \tilde{y}, \\ \left(\frac{1-\delta}{\delta w}\right)^{\frac{(1-\delta)(1-k)}{k}}(Z^*_{nob}(t))^{-\frac{1}{k}}, & \tilde{y} \leq Z^*_{nob}(t) \leq \hat{z}_{nob}, \end{cases}$$

$$l^*_{nob}(t) = \begin{cases} L, & 0 < Z^*_{nob}(t) < \tilde{y}, \\ \left(\frac{1-\delta}{\delta w}\right)^{-\frac{\delta(1-k)-1}{k}}(Z^*_{nob}(t))^{-\frac{1}{k}}, & \tilde{y} \leq Z^*_{nob}(t) \leq \hat{z}_{nob}, \end{cases}$$

$$\pi^*_{nob}(t) = \begin{cases} \frac{\theta}{\sigma}\Big[n_2(n_2-1)B_{21,nob}(Z^*_{nob}(t))^{n_2-1} \\ \qquad + \frac{\delta(1-k)}{(\delta(1-k)-1)^2}\frac{A_1}{\Gamma_1}(Z^*_{nob}(t))^{\frac{1}{\delta(1-k)-1}}\Big], & 0 < Z^*_{nob}(t) < \tilde{y}, \\ \frac{\theta}{\sigma}\Big[n_1(n_1-1)B_{12,nob}(Z^*_{nob}(t))^{n_1-1} + n_2(n_2-1)B_{22,nob}(Z^*_{nob}(t))^{n_2-1} \\ \qquad + \frac{1-k}{k^2}\frac{A_2}{\Gamma_2}(Z^*_{nob}(t))^{-\frac{1}{k}}\Big], & \tilde{y} \leq Z^*_{nob}(t) \leq \hat{z}_{nob}, \end{cases}$$

and the optimal wealth process is

$$X^*_{nob}(t) = \begin{cases} -n_2 B_{21,nob}(Z^*_{nob}(t))^{n_2-1} - \frac{\delta(1-k)}{\delta(1-k)-1}\frac{A_1}{\Gamma_1}(Z^*_{nob}(t))^{\frac{1}{\delta(1-k)-1}} \\ \qquad - \frac{w(\bar{L}-L)-d}{r}, & 0 < Z^*_{nob}(t) < \tilde{y}, \\ -n_1 B_{12,nob}(Z^*_{nob}(t))^{n_1-1} - n_2 B_{22,nob}(Z^*_{nob}(t))^{n_2-1} \\ \qquad + \frac{1-k}{k}\frac{A_2}{\Gamma_2}(Z^*_{nob}(t))^{-\frac{1}{k}} - \frac{w\bar{L}-d}{r}, & \tilde{y} \leq Z^*_{nob}(t) \leq \hat{z}_{nob}. \end{cases}$$

- Case 2. $0 < \hat{z}_{nob} < \tilde{y}$: the optimal consumption-portfolio-leisure strategy defined on the interval $0 < Z^*_{nob}(t) \leq \hat{z}_{nob}$ is

$$c^*_{nob}(t) = L^{-\frac{(1-k)(1-\delta)}{\delta(1-k)-1}}(Z^*_{nob}(t))^{\frac{1}{\delta(1-k)-1}}, \qquad l^*_{nob}(t) = L,$$

$$\pi^*_{nob}(t) = \frac{\theta}{\sigma}\left[n_2(n_2-1)B_{2,nob}(Z^*_{nob}(t))^{n_2-1} + \frac{\delta(1-k)}{(\delta(1-k)-1)^2}\frac{A_1}{\Gamma_1}(Z^*_{nob}(t))^{\frac{1}{\delta(1-k)-1}}\right],$$

and the optimal wealth process is

$$X^*_{nob}(t) = -n_2B_{2,nob}(Z^*_{nob}(t))^{n_2-1} - \frac{A_1}{\Gamma_1}\frac{\delta(1-k)}{\delta(1-k)-1}(Z^*_{nob}(t))^{\frac{1}{\delta(1-k)-1}} - \frac{w(\bar{L}-L)}{r}.$$

E Parameter Definitions

Table E.1: Summary of Input Parameters

**Input Parameters**	**Definitions**
δ	**exponent of consumption in the utility function**
k	**risk aversion related coefficient**
r	**risk-free rate**
μ	**return of the risky asset**
σ	**volatility of the risky asset**
γ	**subjective discount rate**
d	**debt repayment amount**
w	**wage rate**
F	**fixed cost of bankruptcy**
$F+\eta$	**liquidity constraint boundary for the pre bankruptcy part**
α	**flexible cost coefficient of bankruptcy**
$\bar{L}$	**sum of labour and leisure rate**
L	**maximum leisure rate**
